# ﻿New species and records of the genus *Hybos* Meigen (Diptera, Empidoidea, Hybotinae) from Wuyishan National Park, China

**DOI:** 10.3897/zookeys.1172.105952

**Published:** 2023-07-27

**Authors:** Meilin Li, Ding Yang

**Affiliations:** 1 Department of Entomology, College of Plant Protection, China Agricultural University, Beijing 100193, China China Agricultural University Beijing China

**Keywords:** Brachycera, Central China region, East Asia, Empididae, identification key, morphology, new taxon, taxonomy

## Abstract

Wuyishan National Park is well known for its rich biodiversity. Previously, only five species of *Hybos* Meigen, 1803 were known to occur in this region. In this study, 27 species of the genus *Hybos* from Wuyishan National Park are reviewed based on comparative morphological characteristics. Among these, nine species are described as new to science: *Hybosbrevidigitatus***sp. nov.**, *Hybosconstractus***sp. nov.**, *Hyboscurvitibia***sp. nov.**, *Hybosdazhulanus***sp. nov.**, *Hybosfujianensis***sp. nov.**, *Hybosleucopogus***sp. nov.**, *Hyboslongidigitatus***sp. nov.**, *Hybosmodificatus***sp. nov.**, and *Hyboswuyishanus***sp. nov.** Diagnoses, detailed descriptions, remarks, colored illustrations, distributions, and some of the female terminalia characteristics are provided for nine new species. In addition, 13 species of this genus are reported for the first time in Wuyishan National Park. A key to *Hybos* species from Wuyishan National Park is also given.

## ﻿Introduction

The genus *Hybos* Meigen, 1803 belongs to the subfamily Hybotinae (Diptera: Empididae) and is distributed worldwide with 233 known species, of which 28 species are distributed in the Palaearctic Realm and 182 species in the Oriental Realm (Yang et al. 2007; Shamshev et al. 2013, 2015; Wang et al. 2015; Wei et al. 2016; Li et al. 2017; Cao et al. 2018; Kanavalova et al. 2021; Liu et al. 2021; Li et al. 2022). *Hybos* has the following distinctive morphological characters: Rs short; cell cup usually distinctly longer than bm; eyes narrowly but distinctly separated on face; proboscis long spine-like, as long as head or longer, lacking pseudotracheae; hind femur usually strongly thickened with strong ventral bristles (Yang and Yang 2004; Plant 2013). In the past two decades, many taxonomic studies have been carried out on *Hybos*, mainly focusing on the species in China (Yang and Yang 2004; Li et al. 2017; Cao et al. 2018; Li et al. 2022), Thailand (Plant 2013), and Europe (Shamshev et al. 2015; Kanavalova et al. 2021).

Wuyishan National Park is one of the first five national parks officially established in China, with a total area of 1,258 km^2^. It is the intersection of China’s biodiversity hotspot and a well-preserved subtropical upper montane forest, one of the 11 global biodiversity conservation areas in China, and the gene pool of wild animals and plants in the mid-subtropical regions. There were only five known species of *Hybos* here previously: *H.chinensis* Frey, 1953; *H.griseus* Yang & Yang, 1991; *H.jianyangensis* Yang & Yang, 2004; *H.longshengensis* Yang & Yang, 1986; and *H.orientalis* Yang & Yang, 1986 (Yang and Yang 2004).

In this paper, based on the specimens of *Hybos* preserved in the Entomological Museum of China Agricultural University (CAU), 27 species from Wuyishan National Park are reviewed. Among them, nine new species are found: *H.brevidigitatus* sp. nov., *H.constractus* sp. nov., *H.curvitibia* sp. nov., *H.dazhulanus* sp. nov., *H.fujianensis* sp. nov., *H.leucopogus* sp. nov., *H.longidigitatus* sp. nov., *H.modificatus* sp. nov., *H.wuyishanus* sp. nov.; and 13 species are reported for the first time in Wuyishan National Park: *H.anae* Yang & Yang, 2004, *H.ancistroides* Yang & Yang, 1986, *H.basiflavus* Yang & Yang, 1986, *H.bispinipes* Saigusa, 1965, *H.concavus* Yang & Yang, 1991, *H.flaviscutellum* Yang & Yang, 1986, *H.gutianshanus* Yang & Yang, 1995, *H.serratus* Yang & Yang, 1992, *H.uniseta* Yang & Yang, 2004, *H.wangae* Yang, Merz & Grootaert, 2006, *H.wui* Yang & Yang, 1995, *H.xiaohuangshanensis* Yang, Gaimari & Grootaert, 2005, and *H.zhejiangensis* Yang & Yang, 1995 (Yang and Yang 2004; Yang et al. 2005, 2006). Diagnoses, illustrations, and distributions are provided for all species. An identification key to 27 species from Wuyishan National Park has also been constructed.

## ﻿Materials and methods

All specimens for this study were collected from Fujian, China by various entomologists in 2021. The specimens were studied and illustrated using a ZEISS Stemi 2000-c. An image of the lateral view of specimens was taken by connecting the microscope with a Canon 5D camera. Genitalic preparations were made by macerating the apical portion of the abdomen in cold 20% hydroxide (NaOH) for ~ 4–8 hours. The type specimens are deposited in the Entomological Museum of China Agricultural University (**CAU**), Beijing.

The following abbreviations are used in the descriptions: **acr** - acrostichal bristle(s), **ad** - anterodorsal bristle(s), **av** - anteroventral bristle(s), **dc** - dorsocentral bristle(s), **mv** - mesoventral bristle(s), **npl** - notopleural bristle(s), **oc** - ocellar bristle(s), **pd** - posterodorsal bristle(s), **ppn** - postpronotal bristle(s), **prsc** - prescutellar bristle(s), **psa** - postalar bristle(s), **pv** - posteroventral bristle(s), **sc** - scutellar bristle(s).

## ﻿Taxonomy

### ﻿Key to species of *Hybos* from Wuyishan National Park

(This key is used for *Hybos* identification of Wuyishan National Park. The user is urged to confirm all determinations by reference to the detailed descriptions. Caution is also needed as other species in Wuyishan National Park remain undescribed and some of the species will be found elsewhere.)

**Table:** 

1	All legs wholly blackish brown to black excluding tarsomere 1	**2**
–	Legs at least partly yellow to dark yellow excluding tarsomere 1	**8**
2	All legs wholly blackish brown to black	**3**
–	Legs blackish brown, but only tarsomere 1 dark yellow	***H.griseus* Yang & Yang, 1991**
3	R_4+5_ and M_1_ weakly convergent or parallel apically	**4**
–	R_4+5_ and M_1_ divergent apically	**7**
4	R_4+5_ and M_1_ nearly parallel apically	***H.anae* Yang & Yang, 2004**
–	R_4+5_ and M_1_ convergent apically	**5**
5	Mid tarsomere 1 with av at extreme base	***H.leucopogus* sp. nov.**
–	Mid tarsomere 1 only with 2 dorsal bristles, without ventral bristles	**6**
6	Fore tarsomere 2 without ad. Female: Sternite 7 baso-lateral and apico-lateral corner slightly protuberant in lateral view; narrow basally, nearly trapezoid in ventral view	***H.curvitibia* sp. nov.**
–	Fore tarsomere 2 with 1 long subapical ad. Female: Sternite 7 only apico-lateral corner slightly protuberant in lateral view; nearly quadrate in ventral view	***H.modificatus* sp. nov.**
7	Arista bare; hypandrium bifurcated apically	***H.bispinipes* Saigusa, 1965**
–	Arista pubescent; hypandrium with oblique apical margin narrowing towards tip	***H.jianyangensis* Yang & Yang, 2004**
8	Fore or mid tarsomeres 1 or 2 blackish brown to black	**9**
–	Fore and mid tarsomeres 1 or 2 yellow to dark yellow	**15**
9	Arista pubescent	***H.concavus* Yang & Yang, 1991**
–	Arista bare	**10**
10	Hind tarsus yellow to brownish yellow	**11**
–	Hind tarsus blackish brown to blackish	**13**
11	Mid tibia yellow	***H.longshengensis* Yang & Yang, 1986**
–	Mid tibia blackish brown to black	**12**
12	Mid tibia with 1 very long dorsal bristle at base, 1 very long dorsal bristle and 1 ventral bristle at middle; hypandrium with 1 thin finger-like process	***H.ancistroides* Yang & Yang, 1986**
–	Mid tibia with 2 long dorsal bristles on basal 1/2 and 2 ad on apical 1/2; hypandrium with oblique incision	***H.xiaohuangshanensis* Yang, Gaimari & Grootaert, 2005**
13	First flagellomere not elongated, with 2 blackish dorsal hairs; R_4+5_ and M_1_ parallel apically; abdomen short thick	***H.fujianensis* sp. nov.**
–	First flagellomere much elongated, without dorsal hairs; R_4+5_ and M_1_ divergent apically; abdomen long narrow	**14**
14	Fore tibia with 1 ad near middle; mid tibia with 2 very long ad on basal 1/2 and 1 very long av at middle; hind tibia without row of ad and pd	***H.dazhulanus* sp. nov.**
–	Fore tibia with 1 long strong preapical ad; mid tibia with 2 long strong ad on apical 1/2 and 1 long pv at middle; hind tibia with 1 row of short thin ad and pd	***H.wuyishanus* sp. nov.**
15	Fore and mid femora blackish brown to black	**16**
–	Fore and mid femora yellow to dark yellow	**20**
16	Hind tibia with 1 row of short thin pd on apical 1/2	***H.longidigitatus* sp. nov.**
–	Hind tibia without row of pd on apical 1/2	**17**
17	All tibiae mostly blackish brown to black	**18**
–	All tibiae dark yellow to yellow	***H.zhejiangensis* Yang & Yang, 1995**
18	Left surstylus with one curved process and one hook-like process	***H.basiflavus* Yang & Yang, 1986**
–	Left surstylus only with one thin finger-like process	***H.wui* Yang & Yang, 1995**
19	Hind femur blackish brown to black	**20**
–	Hind femur yellow to brownish yellow	**22**
20	Hind tibia with no dorsal hairs at middle	***H.brevidigitatus* sp. nov.**
–	Hind tibia at least with 1 dorsal hair at middle	**21**
21	Hypandrium not furcated apically	***H.chinensis* Frey, 1953**
–	Hypandrium bifurcated apically	**22**
22	Left surstylus trifurcated, right surstylus without furcation	***H.constractus* sp. nov.**
–	Left surstylus bifurcated, right surstylus trifurcated	***H.uniseta* Yang & Yang, 2004**
23	Thorax blackish brown to blackish except mesopleuron brownish yellow	**24**
–	Thorax entirely blackish brown to black	**25**
24	Hypandrium obtuse apically	***H.flaviscutellum* Yang & Yang, 1986**
–	Hypandrium bifurcated apically	***H.wangae* Yang, Merz & Grootaert, 2006**
25	Right surstylus with 3 denticles	***H.serratus* Yang & Yang, 1992**
–	Right surstylus without denticles	**26**
26	Hypandrium with 2 processes of nearly equal width	***H.gutianshanus* Yang & Yang, 1995**
–	Hypandrium with 1 thin left process and 1 wide right process	***H.orientalis* Yang & Yang, 1986**

### ﻿Checklist of *Hybos* in Wuyishan National Park of China

New records in bold

*Hybosanae* Yang & Yang, 2004 (**Fujian**, Guangxi)

*Hybosancistroides* Yang & Yang, 1986 (Guizhou, **Fujian**, Guangxi; Thailand)

*Hybosbasiflavus* Yang & Yang, 1986 (Guizhou, **Fujian**, Guangxi)

*Hybosbispinipes* Saigusa, 1965 (Zhejiang, Hubei, **Fujian**, Taiwan)

*Hybosbrevidigitatus* sp. nov. (Fujian)

*Hyboschinensis* Frey, 1953 (Zhejiang, Guizhou, Fujian, Guangxi)

*Hybosconcavus* Yang & Yang, 1991 (Henan, Hubei, **Fujian**)

*Hybosconstractus* sp. nov. (Fujian)

*Hyboscurvitibia* sp. nov. (Fujian)

*Hybosdazhulanus* sp. nov. (Fujian)

*Hybosflaviscutellum* Yang & Yang, 1986 (Zhejiang, **Fujian**, Guangxi)

*Hybosfujianensis* sp. nov. (Fujian)

*Hybosgriseus* Yang & Yang, 1991 (Zhejiang, Hubei, Fujian)

*Hybosgutianshanus* Yang & Yang, 1995 (Zhejiang, **Fujian**)

*Hybosjianyangensis* Yang & Yang, 2004 (Zhejiang, Guizhou, Fujian)

*Hybosleucopogus* sp. nov. (Fujian)

*Hyboslongidigitatus* sp. nov. (Fujian)

*Hyboslongshengensis* Yang & Yang, 1986 (Fujian, Guangxi)

*Hybosmodificatus* sp. nov. (Fujian)

*Hybosorientalis* Yang & Yang, 1986 (Henan, Fujian, Guangxi)

*Hybosserratus* Yang & Yang, 1992 (Zhejiang, Henan, Sichuan, Yunnan, Guizhou, **Fujian**, Guangxi; Thailand)

*Hybosuniseta* Yang & Yang, 2004 (Sichuan, Zhejiang, **Fujian**)

*Hyboswangae* Yang, Merz & Grootaert, 2006 (**Fujian**, Guangdong)

*Hyboswui* Yang & Yang, 1995 (Zhejiang, **Fujian**)

*Hyboswuyishanus* sp. nov. (Fujian)

*Hybosxiaohuangshanensis* Yang, Gaimari & Grootaert, 2005 (**Fujian**, Guangdong)

*Hyboszhejiangensis* Yang & Yang, 1995 (Zhejiang, **Fujian**)

### ﻿Descriptions

#### ﻿ Hybos anae

Taxon classificationAnimaliaDipteraHybotidae

Yang & Yang, 2004

BB48829F-EEF0-5C0F-8B60-CBCC6EEB616B

Fig. 1

Hybos anae Yang & Yang, 2004: 124, figs 157–160.

##### Type locality.

China: Guangxi, Longsheng.

##### Material examined.

China • 1♂, Fujian, Wuyishan, Yangludaoban; 890 m, 26 April–3 May 2021; Junli Yao (Malaise trap); CAU.

**Figure 1.**
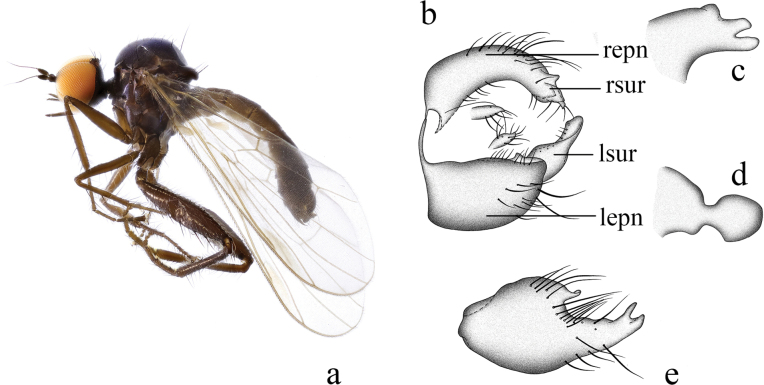
*Hybosanae***a** male habitus, lateral view **b** genitalia, dorsal view **c** right surstylus **d** left surstylus **e** hypandrium, ventral view. Abbreviations: lepn = left epandrial lamella; lsur = left surstylus; repn = right epandrial lamella; rsur = right surstylus.

##### Diagnosis.

Arista short pubescent. Legs nearly entirely blackish brown. Fore tibia with ~ 12 very long ventral hairs apically. R_4+5_ and M_1_ nearly parallel apically. Hypandrium shallowly incised apically, with left subapical corner.

##### Distribution.

China (Fujian, Guangxi).

#### ﻿ Hybos ancistroides

Taxon classificationAnimaliaDipteraHybotidae

Yang & Yang, 1986

E0343629-89A1-50EB-A32B-451BF41DB06D

Fig. 2

Hybos ancistroides Yang & Yang, 1986: 80, fig. 9; Yang and Yang 2004: 126, figs 161–164.

##### Type locality.

China: Guangxi, Jinxiu.

##### Material examined.

China • 16♂♂ 28♀♀, Fujian, Wuyishan, Tongmuguan; 800–900 m, 11 July 2009; Xiaoyan Liu; CAU.

**Figure 2.**
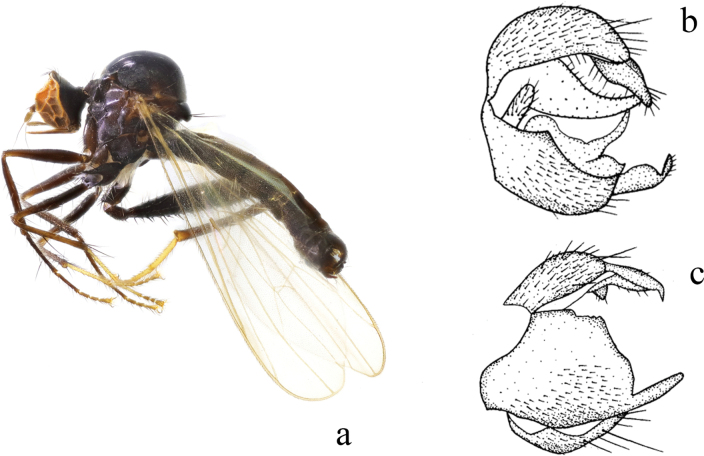
*Hybosancistroides***a** male habitus, lateral view **b** genitalia, dorsal view **c** genitalia, ventral view (from Yang and Yang 1986).

##### Diagnosis.

Arista bare. Legs blackish brown, except mid and hind tarsi, tip of hind femur and base of tibiae yellow, sometimes tip of hind tibia yellow. Hypandrium large and wide, left corner with one finger-like process apically.

##### Distribution.

China (Guizhou, Fujian, Guangxi); Thailand.

#### ﻿ Hybos basiflavus

Taxon classificationAnimaliaDipteraHybotidae

Yang & Yang, 1986

D5E6B56B-1BBF-5D1E-832C-A41EC3346E15

Fig. 3

Hybos basiflavus Yang & Yang, 1986: 77, fig. 5; Yang and Yang 2004: 134, figs 182–185.

##### Type locality.

China: Guangxi, Jin Xiu.

##### Material examined.

China • 1♂ 1♀, Fujian, Wuyishan, Feicuigu; 203 m, 15 April 2021; Ding Yang; CAU. China • 2♂♂ 1♀, Fujian, Wuyishan, Erlichang; 764 m, 10–17 May 2021; Lingfei Peng (Malaise trap); CAU. China • 1♂, Fujian, Wuyishan, Erlichang; 764 m, 25 May–1 June 2021; Lingfei Peng (Malaise trap); CAU. China • 1♂ 1♀, Fujian, Wuyishan, Daanyuan; 520 m, 25 September 2021; Junli Yao (Malaise trap); CAU. China • 1♂, Fujian, Wuyishan, Xianfengling; 1,147 m, 8–15 June 2021; Junlin Yao (Malaise trap); CAU. China • 2♂♂, Fujian, Wuyishan, Fangbanchang; 954 m, 3–10 May 2021; Junli Yao (Malaise trap); CAU.

**Figure 3.**
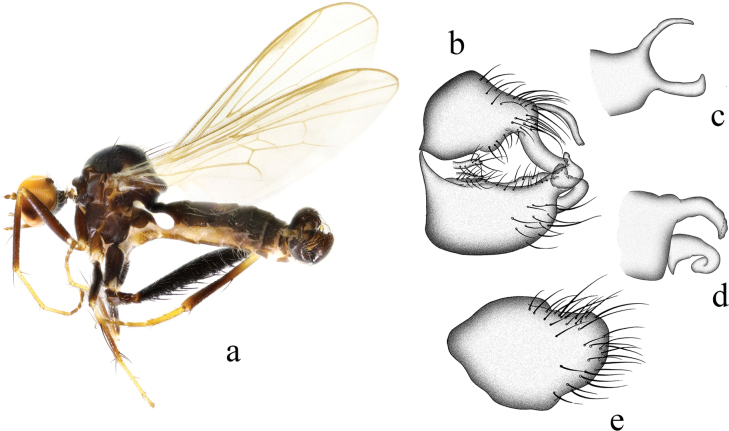
*Hybosbasiflavus***a** male habitus, lateral view **b** genitalia, dorsal view **c** right surstylus **d** left surstylus **e** hypandrium, ventral view.

##### Diagnosis.

Ocellar tubercle with two very short oc and without posterior hairs. Legs mostly brown except base of all tibiae and all tarsomeres 1 and 2 yellow. Hind tibia with one ad at middle, one preapical ad and pd, one av at extreme tip. Left surstylus with one curved process and one hook-like process. Hypandrium large and wide, apical margin obtuse.

##### Distribution.

China (Guizhou, Fujian, Guangxi).

#### ﻿ Hybos bispinipes

Taxon classificationAnimaliaDipteraHybotidae

Saigusa, 1965

A31F5E27-6BB7-54F9-994A-B6E465D6F251

Fig. 4

Hybos (Hybos) bispinipes Saigusa, 1965: 192.
Hybos bispinipes Yang & Yang, 2004: 138, figs 190, 191.

##### Type locality.

China: Taiwan, Tattaka-Oiwake.

##### Material examined.

China • 1♂, Fujian, Wuyishan, Erlichang; 764 m, 8–15 June 2021; Lingfei Peng (Malaise trap); CAU. China • 1♂, Fujian, Wuyishan, Fangbanchang; 954 m, 10–17 May 2021; Junlin Yao (Malaise trap); CAU.

**Figure 4.**
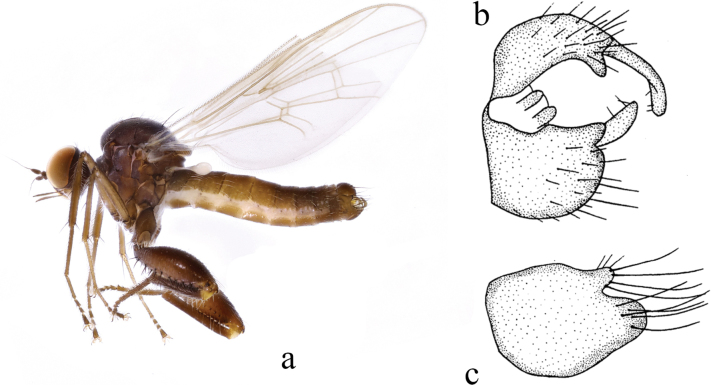
*Hybosbispinipes***a** male habitus, lateral view **b** genitalia, dorsal view **c** hypandrium, ventral view (from Yang and Yang 2004).

##### Diagnosis.

Arista bare. Proboscis nearly as long as head. Legs blackish brown. Hind femur distinctly thickened. Left epandrial lobe large and wide, right surstylus bifurcated with one small process and one rod-like process curved inward. Right epandrial lobe long and narrow. Hypandrium bifurcated apically.

##### Distribution.

China (Zhejiang, Hubei, Fujian, Taiwan).

#### ﻿ Hybos brevidigitatus sp. nov.

Taxon classificationAnimaliaDipteraHybotidae

3CCE04EA-D675-597F-9C5E-34F551F5E399

https://zoobank.org/8DA770E6-9803-4E65-9B6D-0353681145AB

Fig. 5

##### Type material examined.

***Holotype***: China •♂; Fujian, Wuyishan, Feicuigu; 203 m, 15 April 2021; Ding Yang; CAU.

##### Diagnosis.

Arista pubescent. Legs dark yellow, except hind coxae brownish yellow; hind femur blackish brown with blackish tip; hind tibiae dark brown and all tarsomeres 3 and 5 brown. Hypandrium with apico-lateral incision bearing one subtriangular process with long bristles.

**Figure 5.**
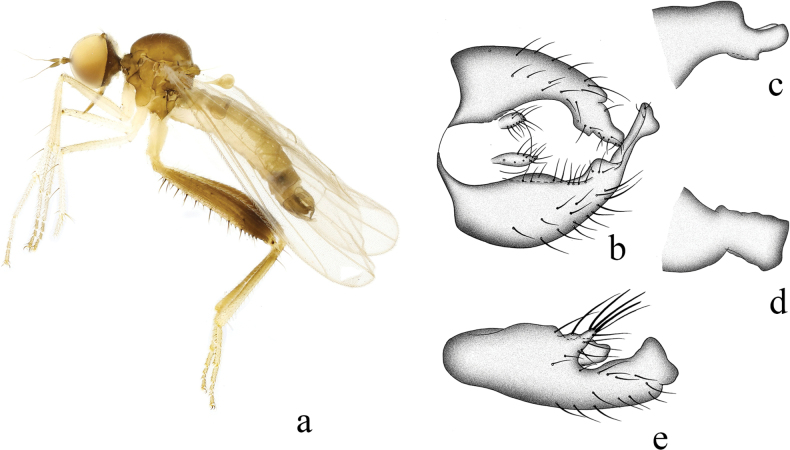
*Hybosbrevidigitatus* sp. nov. **a** male habitus, lateral view **b** genitalia, dorsal view **c** right surstylus **d** left surstylus **e** hypandrium, ventral view.

##### Description.

**Male.** Body length 3.6 mm, wing length 3.7 mm.

***Head*** blackish brown with gray pilosity. Eyes contiguous on frons, dark brown with indistinctly enlarged dorsal facets dark yellow. Hairs and bristles on head blackish brown except postero-ventral surface with partly dark yellow hairs; ocellar tubercle indistinct, with two long oc and two short posterior hairs. Antenna blackish brown; scape without hairs, pedicel with circlet of dark brown subapical hairs; first flagellomere dark brown, much elongated, longer than scape and pedicel combined, without dorsal hairs; arista brown, pubescent, except apical 1/3 thin and bare. Proboscis slightly shorter than head, dark brown. Palpus brownish, with one ventral hair and one apical hair.

***Thorax*** dark brown with gray pilosity. Hairs on thorax brown, bristles dark brown; hairs on mesonotum sparse, slightly long, ppn absent, two npl (posterior npl long strong), uniserial hair-like dc nearly as long as irregularly biserial acr, one long prsc, one psa; scutellum with eight marginal hairs (~ 1/3 as long as sc) and two very long sc. Legs dark yellow, except hind coxae brownish yellow; hind femur blackish brown with blackish tip; hind tibiae dark brown and all tarsomeres 3 and 5 brown. Hairs on legs dark yellow to brown, bristles brownish yellow to blackish brown, but those on coxae dark yellow. Fore femur 1.3× and hind femur 2.6× as wide as mid femur. Fore femur with one row of weak pv ~ 2/5 as long as femur thickness. Mid femur with four or five weak ad on basal 1/2 and one row of pv. Hind femur with three irregular rows of ventral bristles on tubercles (av rather long, short spine-like ventral bristles on distinct tubercles on apical 1/3, pv row interrupted medially with five short pv at base and five very long pv at apex respectively) and one long strong preapical ad. Fore tibia apically with one rather long ad and pd. Mid tibia with two very long ad on basal 2/3; apically with six bristles including one very long pv. Hind tibia with one ad near base. Fore tarsomere 1 with one row of long hair-like ad and pd. Mid tarsomere 1 with one pair of ventral bristles at extreme base. Hind tarsomeres 1 and 2 with several short spine-like ventral bristles. Wing hyaline, slightly tinged brownish, stigma brownish; veins brown, R_4+5_ and M_1_ divergent apically. Squama dark yellow with yellow hairs. Halter yellowish brown with brownish knob.

***Abdomen*** weakly curved downward, brown with pale gray polinosity; hairs and bristles brownish to dark brown except those on hypopygium partly blackish brown. Hypopygium nearly as thick as pregenital segments.

***Male genitalia*.** Left epandrial lamella nearly as wide as right epandrial lamella, with slightly convex inner margin near middle (Fig. 5b); left surstylus nearly trapezoid, wide apically and with one small obtuse subtriangular inner process in lateral view (Fig. 5d). Right epandrial lamella with concave inner margin; right surstylus truncate apically, with one finger-like process (Fig. 5c). Hypandrium ~ 2.8 longer than wide, apically slightly wide, with apico-lateral incision bearing one subtriangular process with long bristles (Fig. 5e).

**Female.** Unknown.

##### Etymology.

This specific name refers to the hypandrium trifurcated with one small figure-like left process.

##### Distribution.

China (Fujian).

##### Remarks.

The new species is similar to *H.wangae* Yang, Merz & Grootaert from Guangdong, but may be separated from the latter by the hind tibia with only one ad near base and right surstylus short in lateral view. In *H.wangae*, the hind tibia does not have bristles at base, and the right surstylus is long in lateral view (Yang et al. 2006).

#### ﻿ Hybos chinensis

Taxon classificationAnimaliaDipteraHybotidae

Frey, 1953

6416B346-8E63-5191-AAA3-B11D26C9448F

Fig. 6

Hybos chinensis Frey, 1953: 64; Yang and Yang 2004: 143, figs 202–207.

##### Type locality.

China: Fujian, “Kwangseh”.

##### Material examined.

China • 1♂ 2♀♀, Fujian, Wuyishan, Liaowangtai; 1,160 m, 8–15 June 2021; Junli Yao(Malaise trap); CAU. China • 1♂, Fujian, Wuyishan, Tongmuguan; 800–900 m, 11 July 2009; Xiaoyan Liu; CAU. China • 1♂ 5♀♀, Fujian, Wuyishan, Dazhulan; 935–1,035 m, 3 June 2009; Xiaoyan Liu; CAU. China • 1♂, Fujian, Wuyishan, Liaowangtai; 1,160 m, 19 April–27 July 2021; Junli Yao (Malaise trap); CAU.

**Figure 6.**
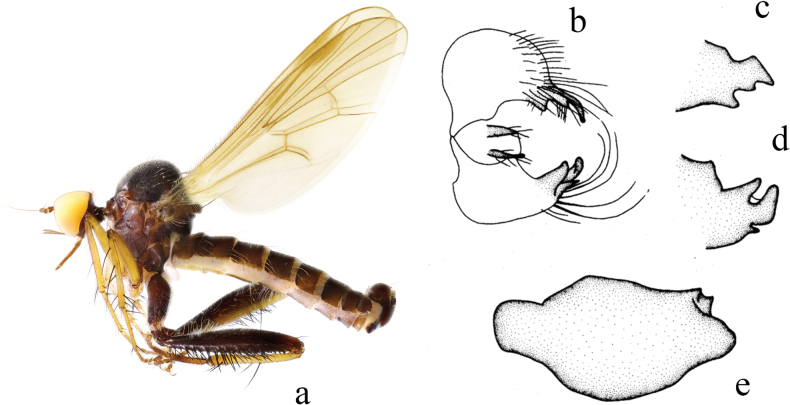
*Hyboschinensis***a** male habitus, lateral view **b** genitalia, dorsal view **c** right surstylus **d** left surstylus **e** hypandrium, ventral view (from Yang and Yang 2004).

##### Diagnosis.

Legs blackish brown except fore and mid trochanters, femora and tarsomeres 1 and 2 and all tibiae yellow; fore and mid tarsomeres 3 and 5 dark brownish yellow; hind tarsus brownish yellow or dark brownish yellow. Hind tibia with single dorsal bristle at middle. Hypandrium large and wide with obtuse apex, left lateral corner with one small process.

##### Distribution.

China (Zhejiang, Guizhou, Fujian, Guangxi).

#### ﻿ Hybos concavus

Taxon classificationAnimaliaDipteraHybotidae

Yang & Yang, 1991

08B37B36-3DA0-59BA-8751-E27D403EC63E

Fig. 7

Hybos concavus Yang & Yang, 1991: 3, fig. 4; Yang and Yang 2004: 145, figs 208–210.

##### Type locality.

China: Hubei, Shennongjia.

##### Material examined.

China •1♂, Fujian, Wuyishan, Yangludaoban; 890 m, 3–10 May 2021; Junli Yao (Malaise trap); CAU.

**Figure 7.**
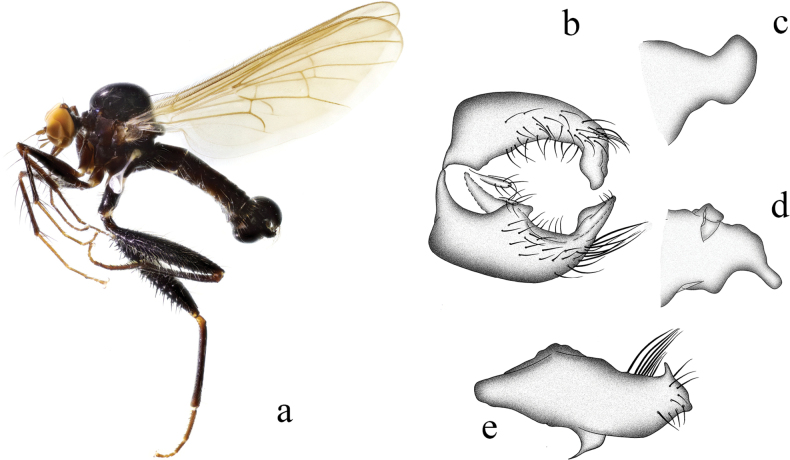
*Hybosconcavus***a** male habitus, lateral view **b** genitalia, dorsal view **c** right surstylus **d** left surstylus **e** hypandrium, ventral view.

##### Diagnosis.

Legs mostly blackish brown to black. Hind tibia apically with single long thin pd and some ad and pd hairs. R_4+5_ and M_1_ nearly parallel apically. Hypandrium long, left lateral margin with nearly arc-shaped concavity apically and one acute process at extreme tip.

##### Distribution.

China (Henan, Hubei, Fujian).

#### ﻿ Hybos constractus sp. nov.

Taxon classificationAnimaliaDipteraHybotidae

73B2F465-0C36-52F7-9AEE-154A07ED90BA

https://zoobank.org/4EB13ACB-0956-452B-9A24-320C766586DD

Fig. 8

##### Type material examined.

***Holotype***: China •♂; Fujian, Wuyishan, Xiaonandingkeng; 1,000 m, 14–27 July 2021; Junli Yao (Malaise trap); CAU.

**Figure 8.**
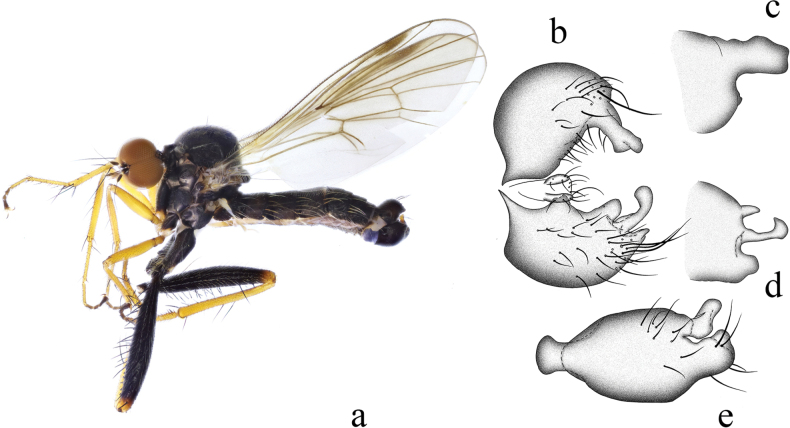
*Hybosconstractus* sp. nov. **a** male habitus, lateral view **b** genitalia, dorsal view **c** right surstylus **d** left surstylus **e** hypandrium, ventral view.

##### Diagnosis.

Ocellar tubercle with two short weak oc and without posterior hairs. Legs mostly dark yellow to brownish yellow. Hind femur distinctly thickened. Hind tibia with one long ad and pd near middle. Hypandrium furcated apically.

##### Description.

**Male.** Body length 4.9 mm, wing length 4.8 mm.

***Head*** black with gray polinosity. Eyes contiguous on frons, blackish brown with distinctly enlarged dorsal facets brownish. Hairs and bristles on head blackish except postero-ventral surface with partly brownish yellow hairs; ocellar tubercle indistinct with two short weak oc and without posterior hairs. Antenna blackish; scape without hairs, pedicel with circlet of blackish subapical hairs; first flagellomere blackish brown except with yellow extreme base, slightly elongated, longer than scape and pedicel combined, without dorsal hairs; arista blackish brown, short pubescent except apical ~1/4 thin and bare. Proboscis nearly as long as head, blackish. Palpus dark brown, with single ventral hair and one apical hair.

***Thorax*** black with gray polinosity. Hairs on thorax blackish except partly brown, bristles black; hairs on mesonotum short, ppn absent, two npl (anterior npl short), uniserial hair-like dc nearly as long as irregularly hexaserial acr, one long prsc, one psa; scutellum with six marginal hairs (2 hairs between sc) and two very long sc. Legs mostly dark yellow to brownish yellow, except base of fore and mid coxae black; hind coxa except extreme tip, knee and femur black; extreme tip of hind femur dark brown and all tarsomeres 3 and 5 blackish brown. Hairs on legs mostly brownish yellow to brown partly dark brown, bristles blackish brown to black, but those on coxae dark yellow; fore and mid femur with partly brownish yellow hairs; hind femur with partly dark yellow hairs. Fore femur 1.3× and hind femur 1.8× as wide as mid femur. Fore femur with one row of weak pv shorter than femur thickness, circlet of preapical bristles including one strong ad. Mid femur with six or seven ad on basal 1/2, one row of weak pv shorter than femur thickness. Hind femur distinctly thickened, 1.7× as wide as hind tibia, with four or five strong ad on apical 1/2, ~ 3 irregular rows of ventral bristles on distinct tubercles mostly long strong. Fore tibia with one weak ad near base, one ad at middle and one strong preapical ad, one preapical av and pv. Mid tibia with one very long strong av near middle, four or five strong ad (1 basal ad very long) on apical 2/3, one long pd near apex; apically with five bristles including one long av. Hind tibia with one long ad and pd at middle, one weak pd near base and one preapical pd. Fore tarsomere 1 with one preapical ad and one pv at extreme base. Mid tarsomere 1 with two av at extreme base (of which one long), three rather strong pd and circlet of preapical bristles. Hind tarsomere 1 with two short spine-like ventral bristles and 3–4 short spine-like av. Wing hyaline, stigma dark brown; veins dark brown, R_4+5_ and M_1_ divergent apically. Squama dark yellow with dark yellow hairs. Halter dark yellow with brownish yellow stem and pale yellow knob.

***Abdomen*** nearly straight, black with pale gray polinosity. Hairs and bristles on abdomen dark yellow to brownish except those on hypopygium mostly brown and partly black. Hypopygium slightly thicker than pregenital segments.

***Male genitalia*.** Left epandrial lamella slightly narrower than right epandrial lamella, with convex inner margin near middle (Fig. 8b); left surstylus trifurcated, with one small subtriangular inner process, one long median process (thick and curved apically), one short wide outer process (nearly trapezoid) (Fig. 8d). Right epandrial lamella with inner margin obliquely subtruncate; right surstylus with one wide finger-like process and weak apical incision (Fig. 8c). Hypandrium ~ 2.1× longer than wide, basally narrow, furcated apically with one wide right process and one narrow curved left process (Fig. 8e).

**Female.** Unknown.

##### Etymology.

This specific name refers to the hypandrium basally narrow.

##### Distribution.

China (Fujian).

##### Remarks.

The new species is similar to *H.chinensis* Frey, but may be separated from the latter by the right surstylus without denticles and hypandrium bifurcated apically. In *H.chinensis*, the right surstylus has the denticles at inner margin, and the hypandrium is not furcated (Yang and Yang 2004).

#### ﻿ Hybos curvitibia sp. nov.

Taxon classificationAnimaliaDipteraHybotidae

E2A637DE-9EA8-5211-AEDE-FF7B303B1A41

https://zoobank.org/32B9D361-3448-4350-ADD3-27AEF653998B

Fig. 9

##### Type material examined.

***Holotype***: China •♂; Fujian, Wuyishan, Dazhulan; 935–1,035 m, 3 July 2009; Xiaoyan Liu; CAU. ***Paratypes***: China • 2♂♂ 17♀♀, same as holotype; CAU. China • 1♂, Fujian, Wuyishan, Dingweizhan; 600 m, 10 July 2009; Xiaoyan Liu; CAU. China • 1♂, Fujian, Wuyishan, Liaowangtai; 1,160 m, 10–17 May 2021; Junli Yao (Malaise trap); CAU.

**Figure 9.**
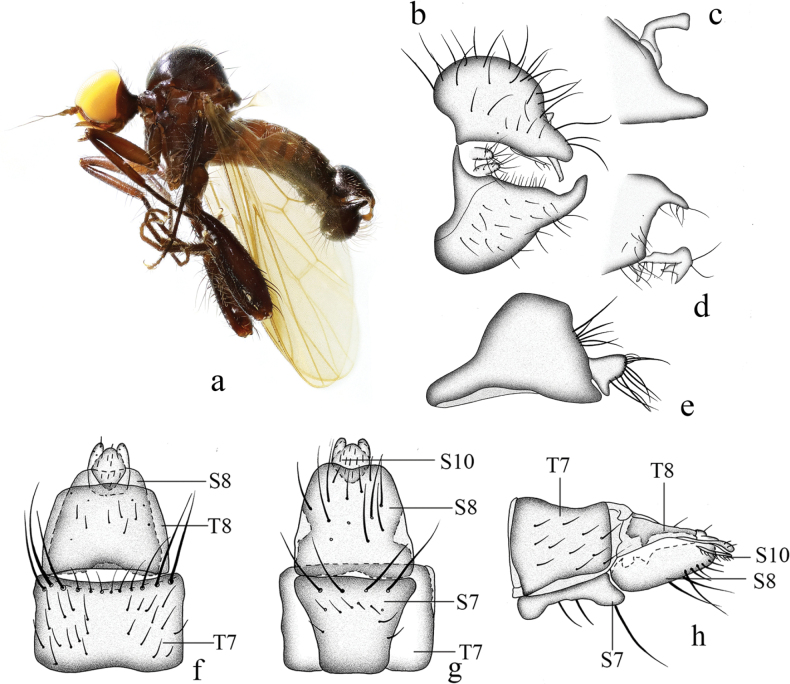
*Hyboscurvitibia* sp. nov. **a** male habitus, lateral view **b** male genitalia, dorsal view **c** right surstylus **d** left surstylus **e** hypandrium, ventral view **f–h** female terminalia **f** dorsal view **g** ventral view **h** lateral view. Abbreviations: S7 = sternite 7; T7 = tergite 7; S8 = sternite 8; T8 = tergite 8; S10 = sternite 10; T10 = tergite 10.

##### Diagnosis.

Legs entirely blackish. Hind tibia slightly curved basally, with single very long ad at middle. Hind femur swollen at middle. R_2+3_ weakly curved, R_4+5_ and M_1_ weakly convergent apically. Hypandrium narrow basally, with truncate apical margin and one small irregular process apically.

##### Description.

**Male.** Body length 3.3–3.7 mm, wing length 3.3–3.5 mm.

***Head*** black, pale gray pilosity. Eyes contiguous on frons, dark yellow, with enlarged upper facets. Hairs and bristles on head black; ocellar tubercle indistinct, with one pair of long oc and two very short posterior hairs. Antenna blackish; scape without hairs; pedicel with circlet of subapical hairs; first flagellomere slightly elongated, without dorsal hairs; arista blackish, very short pubescent except apical ~ 1/4 bare thin. Proboscis blackish; palpus blackish with single hair at extreme tip.

***Thorax*** black, pale grey pilosity; pleuron subshiny. Hairs and bristles black; h absent, two npl (anterior npl short, posterior npl long strong), acr nearly quadriserial, one long posteriormost dc and six hairs anteriad, one long prsc, one long psa; scutellum with six short marginal hairs and two long subapical bristles. Legs entirely black. hairs and bristles on legs black. Fore femur 1.1× and hind femur 1.9× as wide as mid femur. Fore femur with one row of pv hairs (of which some hairs very long). Mid femur with eight very long pv hairs. Hind femur with four long ad at apex, three rows of spine-like v, and with seven or eight very long av hairs on middle 1/3. Fore tibia (except basal portion) with very long hairs. Mid tibia with long hairs and three long ad; apically with one very long av. Hind tibia slightly curved basally, with one long thin ad hair at middle and one long thin ad hair at tip. Fore tarsomeres 1 and 2 with very long hairs; mid tarsomere 1 with one long d at basal 1/3 and one long d at tip, basally with very long hairs. Wing slightly grayish; stigma long, dark brown; veins dark brown, R_2+3_ weakly curved, R_4+5_ and M_1_ weakly convergent apically. Squama dark yellow, bordered with pale hairs. Halter brown.

***Abdomen*** somewhat short thick, weakly curved downward, dark brown with pale gray polinosity except hypopygium black; hairs and bristles dark brown. Hypopygium distinctly thicker than pregenital segments.

***Male genitalia*.** Left epandrial lamella slightly wider than right epandrial lamella (Fig. 9b); left surstylus rather wide, with one short thin subtriangular process, curved inward in lateral view. Right epandrial lamella with inner margin nearly straight (Fig. 9d); right surstylus with one finger-like process, apically narrow, lateral margin bearing one small hook-like process (Fig. 9c). Hypandrium ~ 2× longer than wide, narrow basally, with truncate apical margin and one small irregular process apically (Fig. 9e).

**Female.** Body length 3.6 mm, wing length 3.2 mm. Similar to male, but fore femur with one row of short to long pv hairs. Mid femur without very long pv hairs. Hind femur with four slightly long ad and without very long av hairs. Fore tibia with slightly long hairs. Mid tibia with one long av at middle. Hind tibia straight. Fore tarsomeres 1 and 2 with shorter hairs. Mid tarsomere 1 basally without very long hairs. Terminalia dark brown. Tergite 7 not encircling abdomen, tubular with one acute apico-lateral corner in lateral view (Fig. 9h); 0.6× longer than wide, nearly quadrate with weakly concave basal margin in dorsal view (Fig. 9f). Sternite 7 baso-lateral and apico-lateral corner slightly protuberant in lateral view(Fig. 9h); narrow basally, nearly trapezoid in ventral view (Fig. 9g). Tergite 8 partly membranous, nearly long trapezoid in lateral view (Fig. 9h). Sternite 8 partly membranous, slightly long, and wide in lateral view (Fig. 9h); somewhat quadrate, slightly incised basally nearly as long as wide in ventral view (Fig. 9g). Tergite 10 very weakly sclerotized, hardly differentiated. Sternite 10 membranous on dorsal portion, subtriangular in lateral view (Fig. 9h); wide medially, narrow basally and apically in ventral view (Fig. 9g).

##### Etymology.

This specific name refers to the male hind tibia slightly curved basally.

##### Distribution.

Fujian (Wuyishan).

##### Remarks.

The new species is similar to *H.leucopogus* sp. nov., but may be separated from the latter by the male hind tibia slightly curved basally, and right surstylus with one small hook-like process at lateral margin. In *H.leucopogus* sp. nov., the hind tibia is straight with one row of ad hairs, and the right surstylus does not have the hook-like process. The female of this new species is similar to *H.modificatus* sp. nov., but may be separated from the latter by the sternite 7 baso-lateral and apico-lateral corner slightly protuberant in lateral view and narrow basally, nearly trapezoid in ventral view. In *H.modificatus* sp. nov., the apico-lateral corner of sternite 7 is slightly protuberant in lateral view, and is nearly quadrate in lateral view

#### ﻿ Hybos dazhulanus sp. nov.

Taxon classificationAnimaliaDipteraHybotidae

51A34A65-9492-5928-B29C-265717546156

https://zoobank.org/7DAC6FC7-9290-40F3-B906-E3D32F79755D

Fig. 10

##### Type material examined.

***Holotype***: China •♂; Fujian, Jianyang, Dazhulan; 920 m, 16 April 2021; Ding Yang; CAU. ***Paratypes***: China • 2♂♂ 1♀, same as holotype; CAU. China • 1♂, Fujian, Wuyishan, Xianfengling; 1,147 m, 10–17 May 2021; Junli Yao (Malaise trap); CAU.

**Figure 10.**
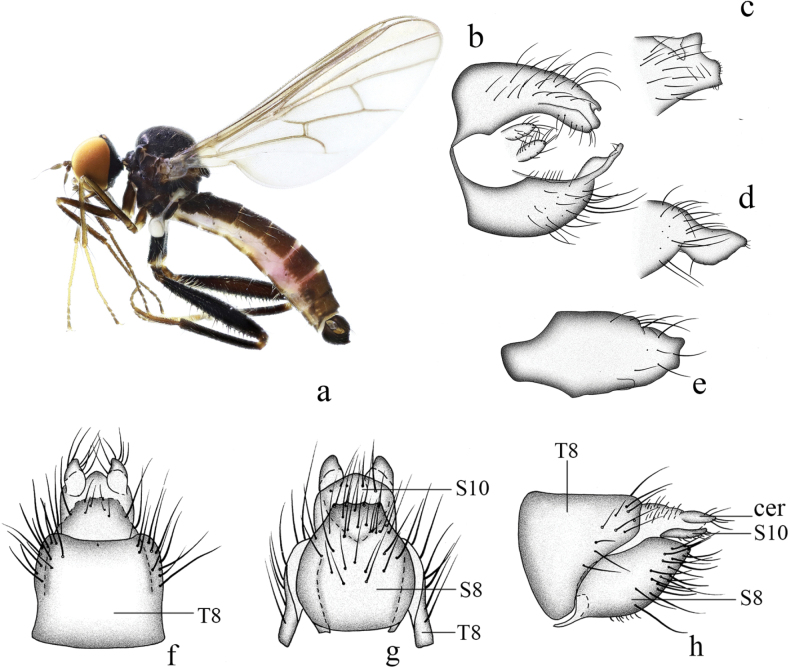
*Hybosdazhulanus* sp. nov. **a** male habitus, lateral view **b** male genitalia, dorsal view **c** right surstylus **d** left surstylus **e** hypandrium, ventral view **f–h** female terminalia **f** dorsal view **g** ventral view **h** lateral view. Abbreviations: cer = cercus; S7 = sternite 7; T7 = tergite 7; S8 = sternite 8; T8 = tergite 8; S10 = sternite 10; T10 = tergite 10.

##### Diagnosis.

First flagellomere much elongated, arista bare. Legs mostly blackish brown to blackish. Abdomen somewhat long narrow. Left surstylus slightly long, narrow apically, with one subtriangular process in lateral view; right surstylus rather wide, with shallow apical incision.

##### Description.

**Male.** Body length 4.7–4.75 mm, wing length 4.0–4.3 mm.

***Head*** black with gray pilosity. Eyes contiguous on frons, dark brown with indistinctly enlarged dorsal facets brownish yellow. Hairs and bristles on head blackish except postero-ventral surface with partly brownish hairs; ocellar tubercle indistinct with two long oc and two short posterior hairs. Antenna blackish; scape without hairs, pedicel with circlet of blackish subapical hairs; first flagellomere dark brown, much elongated, longer than scape and pedicel combined, without dorsal hairs; arista dark brown, bare except apical ~ 1/4 thin. Proboscis shorter than head, blackish. Palpus dark brown, with two ventral hairs and one apical hair.

***Thorax*** black with gray pilosity. Hairs on thorax blackish, bristles black; hairs on mesonotum short to slightly long, ppn absent, two npl (posterior npl long strong), uniserial hair-like dc nearly as long as irregularly quadriserial acr, one very long prsc, one long psa; scutellum with eight marginal hairs (2 hairs located between sc) and two rather long sc. Legs mostly blackish brown to blackish, except fore and mid coxae, tibiae dark brown; hind knee and all tarsi blackish brown; extreme base of hind tibia brownish yellow. Hairs on legs mostly brown to blackish brown, bristles blackish brown to black, but coxae with partly dark yellow hairs; hind femur and tibia with partly brownish hairs. Fore femur 1.2× and hind femur 2.3× as wide as mid femur. Fore femur with one row of weak pv shorter than femur thickness and circlet of preapical bristles. Mid femur with one row of ad on basal 1/2 and one row of pv (middle pv rather long, longer than femur thickness). Hind femur with ~ 3 rows of ventral bristles (av long strong, short spine-like mv on distinct tubercles, long thin pv on basal 1/3), some dense ventral hairs and one preapical ad. Fore tibia with one ad near middle; apically with four bristles including one long ad. Mid tibia with two very long ad on basal 1/2 and one very long av at middle; apically with five bristles including one very long av. Hind tibia apically with one very long thin pd and some dense ventral hairs. Hind tarsomere 1 with one row of short dense spine-like ventral bristles and one short thick apical av. Wing hyaline, stigma brown; veins dark brown, R_4+5_ and M_1_ weakly divergent apically. Squama dark yellow with dark yellow hairs. Halter yellow with pale yellow knob.

***Abdomen*** somewhat long narrow, apically weakly curved downward, blackish with pale gray pilosity; hypopygium nearly as thick as pregenital segments. Hairs and bristles on abdomen brownish yellow to brown except those on hypopygium dark brown.

***Male genitalia*.** Left epandrial lamella nearly as wide as right epandrial lamella, with convex inner margin near middle (Fig. 10b); left surstylus narrowing toward tip, with one obtuse subtriangular inner process in lateral view (Fig. 10d). Right epandrial lamella with concave inner margin near apex; right surstylus short and wide, nearly quadrate, with shallow apical incision (Fig. 10c). Hypandrium ~ 2.6× longer than wide, basally narrow, apically with weak lateral incision (Fig. 10e).

**Female.** Body length 4.3–5.3 mm, wing length 4.8–5.2 mm. Similar to male, but slightly pale. Hind femur and tibia without dense ventral hairs. Terminalia brown. Tergite 8 almost encircling abdomen, sclerotized, wide basally, narrow apically in lateral view (Fig. 10h); 0.9× longer than wide, nearly quadrate with shallow apical incision in dorsal view (Fig. 10f). Sternite 8 bulbous, nearly as long as wide, basally with two indistinct lateral processes in ventral view (Fig. 10g), apically somewhat narrowed. Tergite 10 very weakly sclerotized, hardly differentiated. Sternite 10 weakly sclerotized, finger-like in lateral view (Fig. 10h); somewhat quadrate, narrowing toward tip, nearly as long as wide in ventral view (Fig. 10g). Cerci short, somewhat round, sclerotized at tip (Fig. 10f).

##### Etymology.

This specific name refers to the type locality Dazhulan.

##### Distribution.

China (Fujian).

##### Remarks.

The new species is similar to *H.guanmenshanus* Huo, Zhang & Yang from Hubei, but may be separated from the latter by the hind tibia brownish yellow at extreme base and right surstylus short and wide, nearly quadrate. In *H.guanmenshanus*, the hind tibia is entirely black, and the right surstylus is nearly finger-like apically (Huo et al. 2010).

#### ﻿ Hybos flaviscutellum

Taxon classificationAnimaliaDipteraHybotidae

Yang & Yang, 1986

EC41E09B-EA82-5596-8942-61FC5CCDAB0C

Fig. 11

Hybos flaviscutellum Yang & Yang, 1986: 81, fig. 10; Yang and Yang 2004: 158, figs 241–244.

##### Type locality.

China: Guangxi, Longsheng.

##### Material examined.

China • 1♂ 1♀, Fujian, Wuyishan, Wulichang; 825 m, 8–15 June 2021; Junli Yao (Malaise trap); CAU. China • 1♂ 1♀, Fujian, Wuyishan, Erlichang; 764 m, 8–15 June 2021; Lingfei Peng (Malaise trap); CAU. China • 2♂♂ 1♀, Fujian, Wuyishan, Liaowangtai; 1,160 m, 10–17 May 2021; Junli Yao (Malaise trap); CAU. China • 1♀, Wuyishan, Tongmuguan; 800–900 m, 11 July 2009; Xiaoyan Liu; CAU.

**Figure 11.**
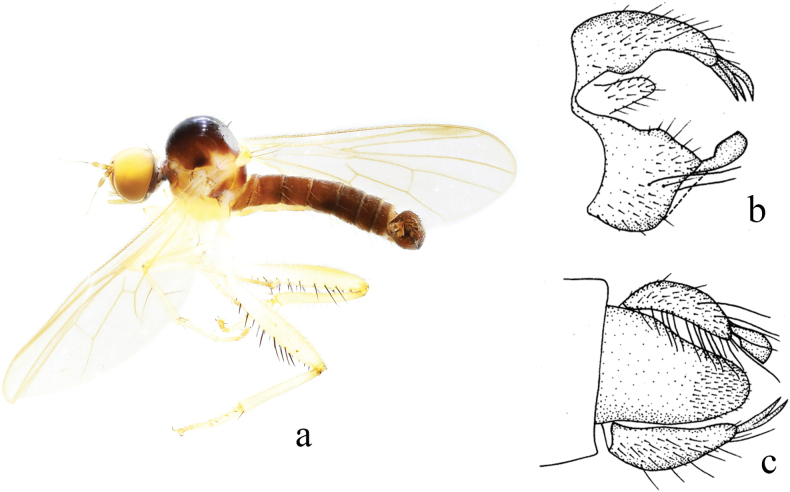
*Hybosflaviscutellum***a** male habitus, lateral view **b** genitalia, dorsal view **c** genitalia, ventral view (from Yang and Yang 1986).

##### Diagnosis.

Legs yellow to brownish yellow. Hind tibia apically with one dorsal bristle and one ventral bristle. Hypandrium large and wide basally, obtuse apically.

##### Distribution.

China (Zhejiang, Fujian, Guangxi).

#### ﻿ Hybos fujianensis sp. nov.

Taxon classificationAnimaliaDipteraHybotidae

85387C69-BF3B-52CD-8EAE-A8C1BCEE0674

https://zoobank.org/1B5AF19E-EF40-430D-8A3A-BC0C5A1B8FE0

Fig. 12

##### Type material examined.

***Holotype***: China •♂; Fujian, Wuyishan, Dingweizhan; 600 m, 10 July 2009; Xiaoyan Liu; CAU. ***Paratype***: China • 1♂, same as holotype; CAU.

##### Diagnosis.

First flagellomere with two blackish dorsal hairs; arista bare. Legs mostly dark brown to blackish brown. Hind tibia apically with long thin pd. R_4+5_ and M_1_ parallel apically. Abdomen short thick.

##### Description.

**Male.** Body length 3.5–4.1 mm. Wing length 4.1–4.3 mm.

***Head*** black with gray pilosity. Eyes contiguous on frons, brownish yellow with slightly enlarged dorsal facets dark yellow. Hairs and bristles on head blackish except postero-ventral surface with partly dark brown hairs; ocellar tubercle distinct with two rather long oc and without posterior hairs. Antenna blackish; scape without hairs, pedicel with circlet of blackish subapical hairs; first flagellomere dark brown, not elongated, nearly as long as scape and pedicel combined, with two blackish dorsal hairs; arista brown, very long and bare except apical ~ 1/4 thin. Proboscis slightly shorter than head, dark brown. Palpus dark brown, with five or six dark brown ventral hairs.

***Thorax*** black with gray pilosity. Hairs on thorax blackish, bristles black; hairs on mesonotum short, ppn absent, two npl (anterior npl rather short, ~ 1/2 as long as posterior one), uniserial hair-like dc nearly as long as irregularly hexaserial acr, two long prsc, one short psa; scutellum with six marginal hairs (~ 1/3 as long as sc) and two long sc. Legs mostly dark brown to blackish brown except all coxae, hind femur blackish and hind knee dark yellow. Hairs on legs mostly dark brown to blackish and partly brown, bristles blackish to black, but those on coxae blackish; hind tibia with partly brown hairs and hind tarsus with brownish yellow ventral hairs. Fore femur 1.2× and hind femur 2.2× as wide as mid femur. Fore femur with one row of weak pv nearly as long as femur thickness. Mid femur with 6–7 strong ad on basal 2/3, one row of pv (middle pv rather long, longer than femur thickness). Hind femur with two ad on apical 2/5, four or five weak pd on basal 1/2 and ~ 3 rows of spine-like ventral bristles on tubercles mostly short except eight long av including four middle av rather long. Fore tibia with one row of short and long ad and some long thin pv hairs on apical 1/2; apically with four bristles including one very long ad and one very long thin pv. Mid tibia with three ad (2 basal ad very long) and two very long av at middle; apically with five bristles including one very long av. Hind tibia apically with long thin pd. Fore tarsomere 1 with two strong pv at extreme base and two very long thin pv hairs at middle. Mid tarsomere 1 with one very long strong av at extreme base. Hind tarsomere 1 with four short spine-like av. Wing hyaline, stigma brownish; veins brownish yellow to brown, R_4+5_ and M_1_ parallel apically. Squama dark yellow with dark yellow hairs. Halter dark yellow with pale yellow knob.

***Abdomen*** short thick with thickened apex including hypopygium slightly thickened, apically distinctly curved downward, dark brown with gray pilosity except basal four segments brown. Hairs and bristles on abdomen brownish yellow or brown except those on hypopygium dark brown.

**Figure 12.**
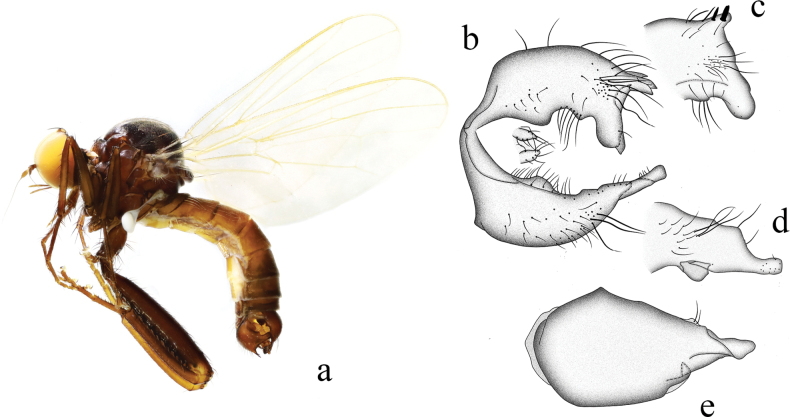
*Hybosfujianensis* sp. nov. **a** male habitus, lateral view **b** genitalia, dorsal view **c** right surstylus **d** left surstylus **e** hypandrium, ventral view.

***Male genitalia*.** Left epandrial lamella slightly narrower than right epandrial lamella (Fig. 12b); left surstylus relatively narrow, obliquely truncate apically and with one finger-like outer process (Fig. 12d). Right epandrial lamella apically distinctly incised at inner margin; right surstylus rather wide, apically with one inner process directed inward and two or three short spine-like bristles on small outer process (Fig. 12c). Hypandrium ~ 2.0× longer than wide, basally rather wide, apically distinctly tapered (Fig. 12e).

**Female.** Unknown.

##### Etymology.

This specific name refers to the type locality Fujian.

##### Distribution.

China (Fujian).

##### Remarks.

The new species is similar to *H.xishuangbannaensis* Yang & Yang from Yunnan, but may be separated from the latter by the mid tibia with three ad and two very long av at middle and right surstylus with 2–3 short spine-like bristles on the small outer process. In *H.xishuangbannaensis*, the mid tibia has two very long dorsal bristles on basal 1/2, and the right surstylus does not have the short spine-like bristles (Yang and Yang 2004).

#### ﻿ Hybos griseus

Taxon classificationAnimaliaDipteraHybotidae

Yang & Yang, 1991

AE5C330C-BB93-5B79-AC20-F40A2ABF435C

Fig. 13

Hybos griseus Yang & Yang, 1991: 5, fig. 6; Yang and Yang 2004: 163, figs 255, 256.

##### Type locality.

China: Hubei, Tongshan.

##### Diagnosis.

Legs blackish brown except tarsomere 1 dark yellow. Hind tibia apically with one dorsal bristle and one ventral bristle. Hypandrium large and wide, bifurcated apically.

**Figure 13.**
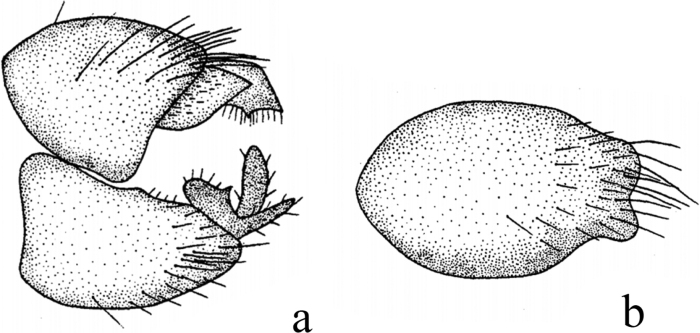
*Hybosgriseus***a** male habitus, lateral view **b** hypandrium, ventral view (from Yang and Yang 1991).

##### Distribution.

China (Zhejiang, Hubei, Fujian).

#### ﻿ Hybos gutianshanus

Taxon classificationAnimaliaDipteraHybotidae

Yang & Yang, 1995

C23C02A6-07DE-5433-AD0C-CF7404EF9393

Fig. 14

Hybos gutianshanus Yang & Yang, 1995: 237, fig. 5; Yang and Yang 2004: 169, figs 268, 269.

##### Type locality.

China: Zhejiang: Gutianshan.

##### Material examined.

China • 4♂♂ 15♀♀, Fujian, Wuyishan, Dazhulan; 935–1,035 m, 3 July 2009; Xiaoyan Liu; CAU.

**Figure 14.**
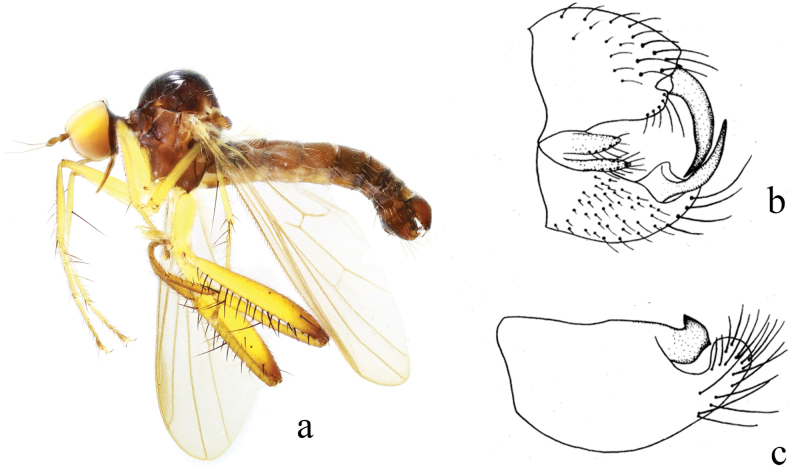
*Hybosgutianshanus***a** male habitus, lateral view **b** genitalia, dorsal view **c** hypandrium, ventral view (from Yang and Yang 1995).

##### Diagnosis.

Arista short pubescent. Legs mostly yellow except tarsomeres 3 and 5 brownish yellow to blackish. Hind tibia with one outer dorsal bristle and one dorsal bristle at middle, apically with three bristles. Hypandrium long and wide.

##### Distribution.

China (Zhejiang, Fujian).

#### ﻿ Hybos jianyangensis

Taxon classificationAnimaliaDipteraHybotidae

Yang & Yang, 2004

39EE7D91-2F5C-5B96-9359-832C49FCE019

Fig. 15

Hybos jianyangensis Yang & Yang, 2004: 178, figs 288–291.

##### Type locality.

China: Fujian, Jianyang.

##### Diagnosis.

Arista short pubescent. Legs all black. Hind tibia without distinct bristles. Hypandrium nearly quadrate, with one thick apical process.

**Figure 15.**
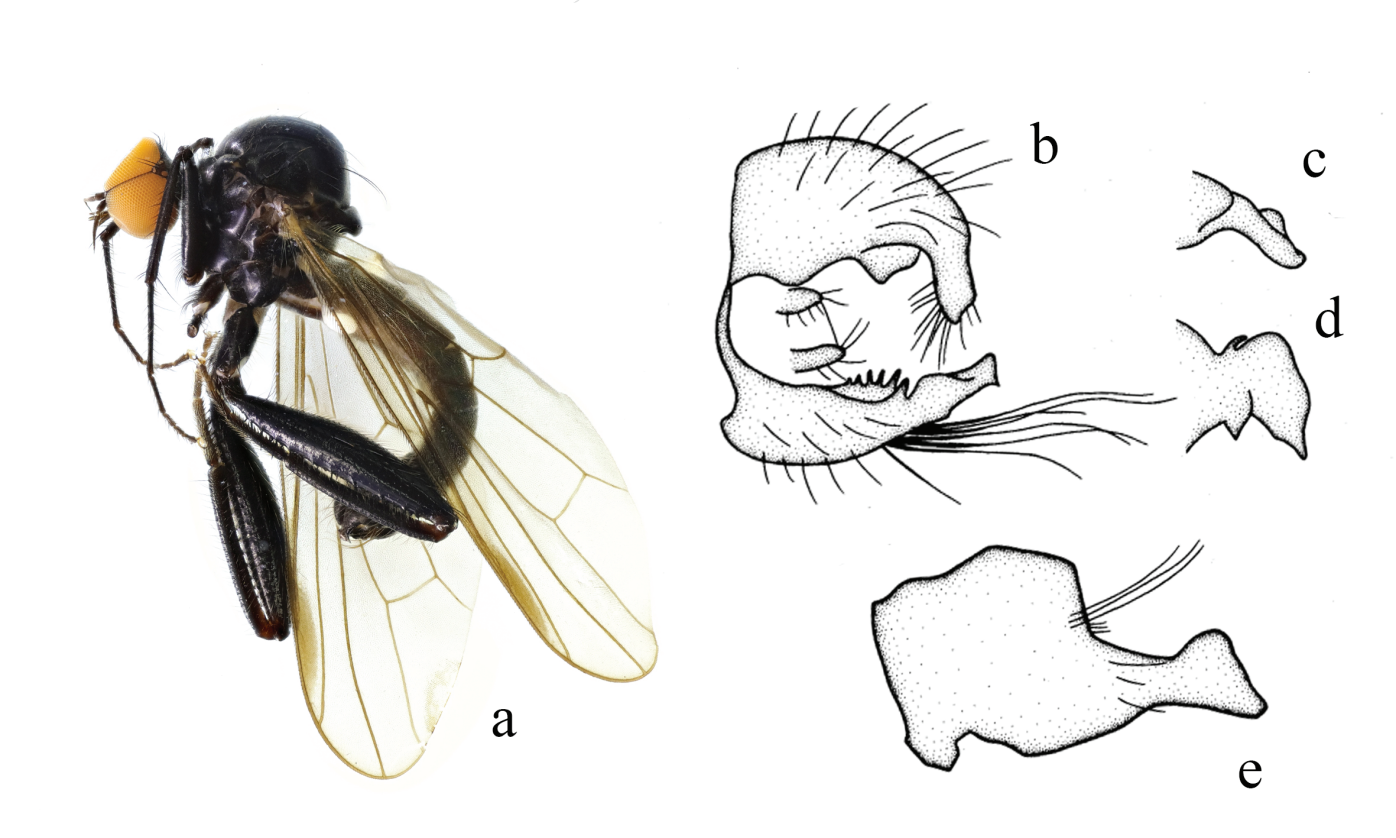
*Hybosjianyangensis***a** male habitus, lateral view **b** genitalia, dorsal view **c** right surstylus **d** left surstylus **e** hypandrium, ventral view (from Yang and Yang 2004).

##### Distribution.

China (Zhejiang, Guizhou, Fujian).

#### ﻿ Hybos leucopogus sp. nov.

Taxon classificationAnimaliaDipteraHybotidae

0973EA52-9DB3-541B-8D24-198C50328CFB

https://zoobank.org/CB24FAED-116A-4740-B38C-384EFB7D6AEE

Fig. 16

##### Type material examined.

***Holotype***: China • ♂; Fujian, Wuyishan, Yangludaoban; 890 m, 7–25 May 2021; Junli Yao (Malaise trap); CAU.

##### Diagnosis.

Legs entirely black. Hind femur distinctly thickened. Hind tibia with one row of ad hairs and four pd hairs on basal ½. R_2+3_ weakly curved, R_4+5_ and M_1_ weakly convergent apically. Hypandrium narrow basally, bifurcated apically.

##### Description.

**Male.** Body length 5.7 mm, wing length 5.4 mm.

***Head*** black with gray pilosity. Eyes contiguous on frons, dark brown with slightly enlarged dorsal facets brownish. Hairs and bristles on head blackish except postero-ventral surface with partly brown hairs; ocellar tubercle indistinct with two oc and with two short posterior hairs. Antenna blackish; scape without hairs, pedicel with circlet of blackish subapical hairs. Proboscis slightly longer than head, dark brown. Palpus blackish brown, with three blackish brown ventral hairs.

***Thorax*** black with gray pilosity. Hairs on thorax blackish, bristles black; hairs on mesonotum rather short, ppn absent, two npl (anterior npl short), uniserial hair-like dc nearly as long as irregularly quadriserial acr, one long prsc, one slightly long psa; scutellum with eight marginal hairs (4 hairs located between sc) and two long sc. Legs entirely black. Hairs on legs dark brown to blackish, bristles blackish to black, but those on coxae brown to dark brown; hind tibia with partly brown apical hairs. Fore femur 1.4× and hind femur 2.3× as wide as mid femur. Fore femur with one row of pv, as long as femur thickness. Mid femur with one row of long av and pv hairs (much longer than femur thickness). Hind femur distinctly thickened, 2.6× as wide as hind tibia, with three long strong ad on apical ½, ~ 3 rows of ventral bristles (av long strong, short spine-like mv on distinct tubercles, pv on basal ½ and very long bristle-like outer pv hairs on apical 1/3. Fore tibia with some long pv hairs at middle; apically with four bristles including one very long strong ad. Mid tibia with one very long ad and three av hairs at middle (of which one long strong); apically with five bristles including one very long av and one long pv. Hind tibia with one row of ad hairs and four pd hairs on basal ½. Fore tarsomere 1 with some long to very long hairs. Mid tarsomere 1 with one av at extreme base and two long ad hairs. Hind tarsomere 1 with two short spine-like av, tarsomeres 1 and 2 with one row of short spine-like ventral bristles. Wing hyaline, stigma brown; veins brown to dark brown, R_2+3_ weakly curved, R_4+5_ and M_1_ weakly convergent apically. Squama dark yellow with dark yellow hairs. Halter dark yellow with pale yellow knob.

***Abdomen*** apically weakly curved downward, black with gray pilosity; hairs and bristles brownish yellow and dark brown except those on hypopygium black. Hypopygium distinctly thicker than pregenital segments.

**Figure 16.**
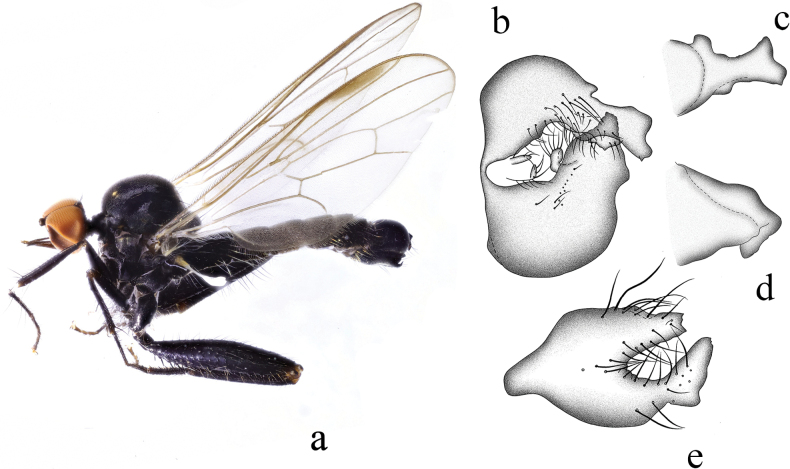
*Hybosleucopogus* sp. nov. **a** male habitus, lateral view **b** genitalia, dorsal view **c** right surstylus **d** left surstylus **e** hypandrium, ventral view.

***Male genitalia*.** Left epandrial lamella wider than right epandrial lamella (Fig. 16b); left surstylus very wide, narrow apically, inner margin with one small, curved process bearing long hairs in lateral view (Fig. 16d). Right epandrial lamella with concave inner margin near apex; right surstylus slightly narrow, apical margin with incision at middle, with one small trapezoid outer process and one small subtriangular inner process (Fig. 16c). Hypandrium ~ 1.7× longer than wide, rather narrow basally, bifurcated apically, with one straight and thick process (which has the truncate apical margin weakly serrate) and one long curved process (which is somewhat narrow medially and much widened apically) (Fig. 16e).

**Female.** Unknown.

##### Etymology.

This specific name refers to all tibiae, and fore and mid tarsi with some hairs.

##### Distribution.

China (Fujian).

##### Remarks.

The new species is similar to *H.modificatus* sp. nov., but may be separated from the latter by the hind tibia straight and hypandrium bifurcated apically. In *H.modificatus* sp. nov., the hind tibia is slightly curved basally, and the hypandrium is not furcated.

#### ﻿ Hybos longidigitatus sp. nov.

Taxon classificationAnimaliaDipteraHybotidae

FA0567FC-C53E-5154-BC9D-8118F2CA93D1

https://zoobank.org/FC1ECAC2-9B76-459A-86BD-E68435B78DC1

Fig. 17

##### Type material examined.

***Holotype***: China •♂; Fujian, Wuyishan, Liaowangtai; 1,160 m, 10–17 May 2021; Junli Yao (Malaise trap); CAU. ***Paratype***: China • 1♀, Fujian, Wuyishan, Wulichang; 825 m, 1–8 June 2021; Junli Yao (Malaise trap); CAU.

##### Diagnosis.

Arista bare. Legs mostly yellow to dark yellow except hind femur with brownish black tip and all tarsomeres 3 and 5 dark brown. Hind tibia with one row of short thin pd on apical 1/2. Hypandrium with apico-lateral incision bearing one long thin finger-like process.

##### Description.

**Male.** Body length 3.7 mm, wing length 3.1 mm.

***Head*** black with gray pilosity. Eyes contiguous on frons, brown with slightly enlarged dorsal facets brownish yellow. Hairs and bristles on head blackish except postero-ventral surface with partly dark yellow hairs; ocellar tubercle distinct with two very long oc and two very short hairs. Antenna blackish brown; scape without hairs, pedicel with circlet of blackish brown subapical hairs; first flagellomere dark brown, slightly elongated, longer than scape and pedicel combined, without dorsal hairs; arista dark brown, bare, except apical ~ 1/3 thin. Proboscis slightly shorter than head, blackish brown. Palpus brown, with one apical hair and one ventral hair.

***Thorax*** black with gray pilosity except pleuron dark brown or blackish brown. Hairs on thorax brown, bristles blackish brown; hairs on mesonotum short to slightly long, ppn absent, two npl (posterior npl very long), uniserial hair-like dc nearly as long as irregularly quadriserial acr, one very long prsc, one long psa; scutellum with six marginal hairs (~ 1/3 as long as sc) and two very long sc. Legs mostly yellow to dark yellow, except hind femur with brownish black tip and all tarsomeres 3 and 5 dark brown. Hairs on legs brownish to dark brown, bristles dark brown to blackish, but those on coxae dark yellow (coxae rarely with partly brownish bristles); hind tarsi with partly brownish yellow hairs. Fore femur 1.5× and hind femur 2.0× as wide as mid femur. Fore femur with one row of weak pv ~ 1/2 as long as femur thickness. Mid femur with one row of long thin pv (middle pv rather long, much longer than femur thickness) and one preapical ad. Hind femur with three irregular rows of ventral bristles (5–6 mostly rather long av along entire length, short spine-like mv on tubercles, dense mv on apical 1/3, long thin pv on apical 1/2, long hair-like pv on apical 1/3). Fore tibia apically with three or four bristles including one very long posterior bristle. Mid tibia with two strong ad on basal 1/2, one very long av at extreme tip. Hind tibia with one row of short thin pd on apical 1/2. Fore tarsomere 1 with three very long thin posterior bristles. Hind tarsomere 1 with one row of short spine-like ventral bristles and one short thick av at extreme tip. Wing hyaline, slightly tinged brownish, stigma brown; veins dark brown, R_4+5_ and M_1_ divergent apically. Squama dark yellow with dark yellow hairs. Halter brownish yellow with pale yellow knob.

***Abdomen*** apically weakly curved downward, blackish brown with pale gray pilosity. Hairs and bristles on abdomen brownish to blackish brown. Hypopygium nearly as thick as pregenital segments.

**Figure 17.**
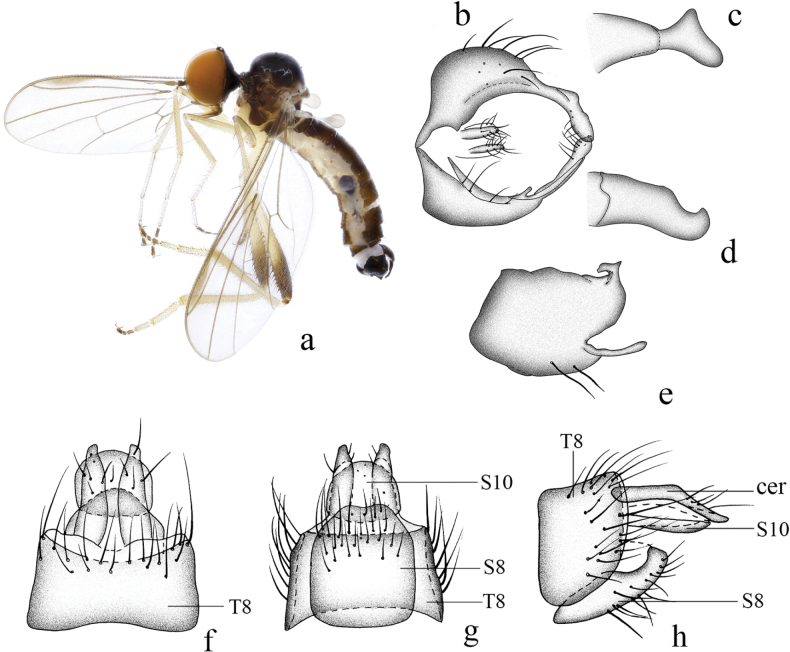
*Hyboslongidigitatus* sp. nov. **a** male habitus, lateral view **b** male genitalia, dorsal view **c** right surstylus **d** left surstylus **e** hypandrium, ventral view **f–h** female terminalia **f** dorsal view **g** ventral view **h** lateral view. Abbreviations: cer = cercus; S7 = sternite 7; T7 = tergite 7; S8 = sternite 8; T8 = tergite 8; S10 = sternite 10; T10 = tergite 10.

***Male genitalia*.** Left epandrial lamella narrow than right epandrial lamella, with concave inner margin (Fig. 17b); left surstylus long narrow, finger-like, with hook-like apically (Fig. 17d). Right epandrial lamella with concave inner margin; right surstylus long, narrow at middle, wide and obtuse apically (Fig. 17c). Hypandrium ~ 1.6× longer than wide, with apico-lateral incision bearing one long thin finger-like process (Fig. 17e).

**Female.** Body length 3.6 mm, wing length 3.9 mm. Similar to male, but mid femur with shorter pv. Terminalia blackish brown. Tergite 8 almost encircling abdomen, sclerotized, wide basally, nearly semicircular apically in lateral view (Fig. 17h); 0.6× longer than wide, somewhat quadrate, basal and apical margin membranous with shallow incision in dorsal view (Fig. 17f). Sternite 8 hook-like, membranous at apico-lateral margin in lateral view (Fig. 17h); 1.2× longer than wide, apical margin slightly narrowed with shallowly incised in ventral view (Fig. 17g). Tergite 10 very weakly sclerotized, hardly differentiated. Sternite 10 membranous on dorsal portion, acute apically, finger-like in lateral view (Fig. 17h); 0.8× longer than wide, rounded in ventral view (Fig. 17g). Cerci very long, curved downward, sclerotized at tip in lateral view (Fig. 17h).

##### Etymology.

This specific name refers to the hypandrium bifurcated apically with one long figure-like process.

##### Distribution.

China (Fujian).

##### Remarks.

The new species is similar to *H.bigeniculatus* Yang & Yang from Hubei, but may be separated from the latter by the hind femur dark yellow with only brownish black tip and all tarsomeres 3 and 5 dark brown. In *H.bigeniculatus*, the hind femur is blackish brown, and all tarsomeres 3 and 5 are yellow (Yang and Yang 2004).

#### ﻿ Hybos longshengensis

Taxon classificationAnimaliaDipteraHybotidae

Yang & Yang, 1986

5A345552-9F69-5FDA-B118-F7698A10CC65

Fig. 18

Hybos longshengensis Yang & Yang, 1986: 78, fig. 6; Yang and Yang 2004: 187, figs 312–315.

##### Type locality.

China: Guangxi, Longsheng.

##### Material examined.

China • 1♂ 1♀, Fujian, Wuyishan, Wulichang; 825 m, 8–15 June 2021; Junli Yao (Malaise trap); CAU. China • 1♂, Fujian, Wuyishan, Liaowangtai; 1,160 m, 10–17 May 2021; Junli Yao (Malaise trap); CAU. China • 1♂ 4♀♀, Fujian, Wuyishan, Tongmucun, Baihuxi; 736 m, 22 June 2021; Xiaodong Cai; CAU.

**Figure 18.**
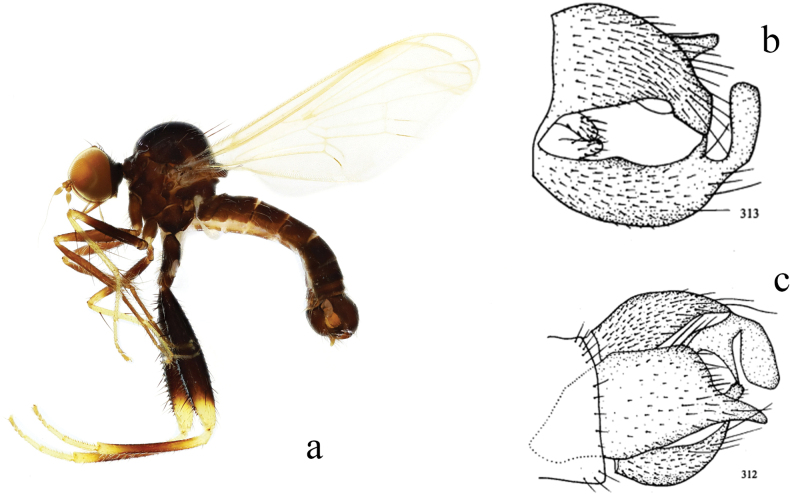
*Hyboslongshengensis***a** male habitus, lateral view **b** genitalia, dorsal view **c** genitalia, ventral view (from Yang and Yang 1986).

##### Diagnosis.

Arista bare, very long. Legs blackish brown, except mid tibia, tip of hind femur, tip and base of tibiae and all tarsi yellow. Hypandrium large and wide, right apico-lateral corner extending into one process.

##### Distribution.

China (Fujian, Guangxi).

#### ﻿ Hybos modificatus sp. nov.

Taxon classificationAnimaliaDipteraHybotidae

F2FF1EE9-2FA6-5757-97CD-B72B170CEA9E

https://zoobank.org/21C151B9-48AD-42CE-9318-6E428D3C72B3

Fig. 19

##### Type material examined.

***Holotype***: China • ♂; Fujian, Wuyishan, Tongmucun, Guadun; 1,033 m, 22 June 2021; Xiaodong Cai; CAU. ***Paratypes***: China • 9♀♀, same as holotype; CAU. China • 10♀♀, Fujian, Wuyishan, Tongmucun, Baihuxi; 736 m, 22 June 2021; Xiaodong Cai; CAU.

##### Diagnosis.

Legs entirely black. Hind tibia with some ad and pd hairs (long pd near apex). Fore tarsomere 2 with one long subapical ad. R_2+3_ curved, R_4+5_ and M_1_ convergent apically. Hypandrium nearly quadrate, with slightly incised apical margin.

##### Description.

**Male.** Body length 3.6 mm.

***Head*** black with gray pilosity. Eyes contiguous on frons, dark brown with distinctly enlarged dorsal facets brownish yellow. Hairs and bristles on head blackish except postero-ventral surface with partly dark brown hairs; ocellar tubercle indistinct with two long oc and two short posterior hairs. Antenna blackish; scape without hairs, pedicel with circlet of blackish subapical hairs; first flagellomere blackish brown, not elongated or slightly elongated, nearly as long as or longer than scape and pedicel combined, without dorsal hairs; arista blackish brown, rather long and short pubescent except apical ~ 1/4 thin and bare. Proboscis slightly shorter than head, blackish. Palpus dark brown, with one ventral hair and one apical hair.

***Thorax*** black with gray pilosity. Hairs on thorax blackish, bristles black; hairs on mesonotum short, ppn absent, two npl (anterior npl rather short), uniserial hair-like dc nearly as long as irregularly hexaserial acr, two long prsc, one psa; scutellum with six short marginal hairs (~ 1/4 as long as sc) and two very long sc. Legs entirely black. Hairs on legs mostly blackish brown to blackish, bristles blackish to black, but those on coxae blackish brown and hind coxae with partly brown, long bristles. Fore femur 1.3× and hind femur 2.2× as wide as mid femur. Fore femur with one row of pv longer than femur thickness. Mid femur with four or five long ad on basal 1/2 and one row of pv (middle pv very long, much longer than femur thickness). Hind femur with three ad and ~ 3 rows of spine-like ventral bristles (av rather strong, very short dense mv on distinct tubercles, pv long thin). Fore tibia with one row of ad and pv (long ad and pv on apical 1/3); apically with three or four bristles including one very long ad and pv. Mid tibia with three long strong ad on basal 2/3 and two long av on apical 1/2. Hind tibia with some ad and pd hairs (long pd near apex). Fore tarsomere 1 with two long ad, three or four long pv hairs and three apical bristles including one long subapical ad. Fore tarsomere 2 with one long subapical ad. Mid tarsomere 1 with two ad on apical 1/2. Hind tarsomeres 1 and 2 with one row of short dense spine-like ventral bristles. Wing hyaline, stigma dark brown; veins dark brown, R_2+3_ curved, R_4+5_ and M_1_ convergent apically. Squama dark yellow with dark yellow hairs. Halter dark yellow with brownish yellow stem and pale yellow knob.

***Abdomen*** straight, black with gray pilosity; hypopygium distinctly thicker than pregenital segments. Hairs and bristles on abdomen blackish brown to black, but basally with some brownish hairs and bristles.

**Figure 19.**
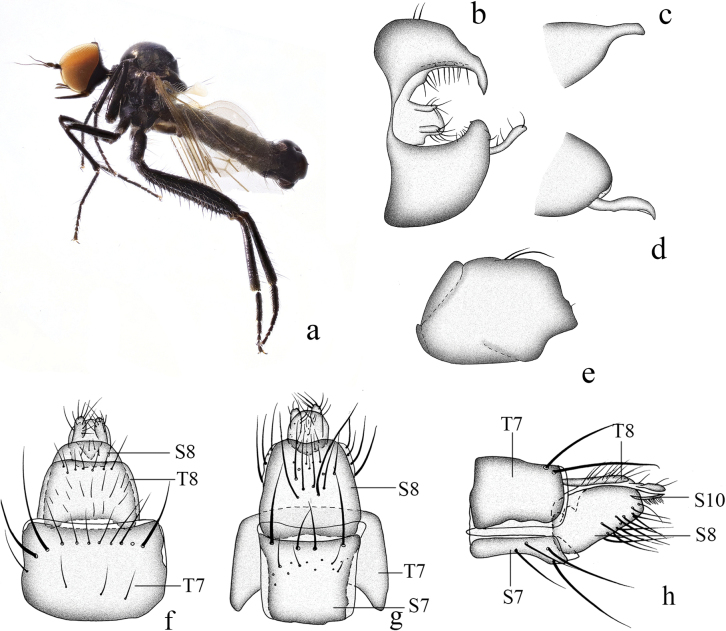
*Hybosmodificatus* sp. nov. **a** male habitus, lateral view **b** genitalia, dorsal view **c** right surstylus **d** left surstylus **e** hypandrium, ventral view **f–h** female terminalia **f** dorsal view **g** ventral view **h** lateral view. Abbreviations: S7 = sternite 7; T7 = tergite 7; S8 = sternite 8; T8 = tergite 8; S10 = sternite 10; T10 = tergite 10.

***Male genitalia*.** Left epandrial lamella and right epandrial lamella widely separated, slightly wide (Fig. 19b); left surstylus obtuse apically, with one long finger-like curved process. Right epandrial lamella very wide, with weakly convex inner margin near base (Fig. 19d); right surstylus long thin, narrow, and acute apically, directed inward in lateral view (Fig. 19c). Hypandrium ~ 1.3× longer than wide, nearly quadrate, with slightly incised apical margin (Fig. 19e).

**Female.** Body length 4.0–4.8 mm, wing length 3.4–4.2 mm. Similar to male, but slightly bigger. Scutellum with six long marginal hairs (~ 1/2 as long as sc). Mid tibia with some long hairs. Hind tibia with one dorsal bristle on apical 1/3. Hind tarsomere 1 with some slightly long dorsal hairs. Terminalia black with some strong black bristles. Tergite 7 not encircling abdomen, tubular in lateral view (Fig. 19h); 0.7× longer than wide, nearly quadrate with weakly convex basal margin, membranous at apical margin in dorsal view (Fig. 19f). Sternite 7 apico-lateral corner slightly protuberant in lateral view (Fig. 19h); nearly quadrate with four strong bristles at apical margin, membranous at apical margin and each side, nearly as long as wide in ventral view (Fig. 19g). Tergite 8 long thin with dense bristles in lateral view (Fig. 19h); weakly sclerotized and nearly trapezoid in dorsal view (Fig. 19f). Sternite 8 partly membranous, large, and wide in lateral view (Fig. 19h); somewhat quadrate, slightly incised apically nearly as long as wide in ventral view (Fig. 19g). Tergite 10 very weakly sclerotized, hardly differentiated. Sternite 10 membranous on dorsal portion, subtriangular in lateral view (Fig. 19h); rounded, slightly acute basally in ventral view (Fig. 19g).

##### Etymology.

This specific name refers to the fore tarsomere 2 with one long subapical ad.

##### Distribution.

China (Fujian).

##### Remarks.

The new species is similar to *H.obtusatus* Yang & Grootaert from Guizhou and Guangdong, but may be separated from the latter by the hind tibia with some ad and pd hairs, and R_4+5_ and M_1_ convergent apically. In *H.obtusatus*, the hind tibia does not have ad and pd hairs, and R_4+5_ and M_1_ are parallel apically (Yang and Grootaert 2005).

#### ﻿ Hybos orientalis

Taxon classificationAnimaliaDipteraHybotidae

Yang & Yang, 1986

53D70A9A-39F1-527C-B211-3ED6CC39A43A

Fig. 20

Hybos orientalis Yang & Yang, 1986: 82, fig. 11; Yang and Yang 2004: 201, figs 346–351.

##### Type locality.

Guangxi (Longsheng), Fujian (Jianyang).

##### Diagnosis.

Legs mostly yellow. Mid tibia with one very long dorsal bristle at basal 1/3, one very long dorsal bristle and one ventral bristle at middle; apically with three bristles. Left surstylus hook-like.

**Figure 20.**
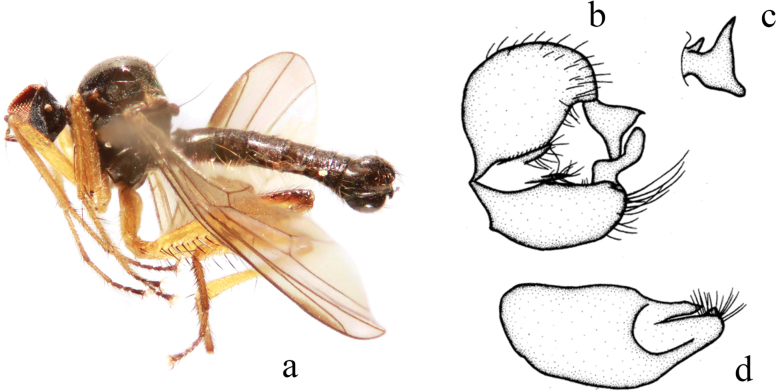
*Hybosorientalis***a** male habitus, lateral view **b** genitalia, dorsal view **c** right surstylus **d** hypandrium, ventral view (from Yang and Yang 2004).

##### Distribution.

China (Henan, Fujian, Guangxi).

#### ﻿ Hybos serratus

Taxon classificationAnimaliaDipteraHybotidae

Yang & Yang, 1992

8D54BFBF-55C9-5968-A11D-D77E9F69D54D

Fig. 21

Hybos serratus Yang & Yang, 1992: 1089, fig. 1; Yang and Yang 2004: 210, figs 372, 373.

##### Type locality.

China: Sichuan, Xichang.

##### Material examined.

China • 2♂♂ 16♀♀, Fujian, Wuyishan, Tongmuguan; 800–900 m, 11 July 2009; Xiaoyan Liu; CAU. China • 1♂ 1♀, Fujian, Wuyishan, Tongmucun, Guadun; 1,033 m, 22 June 2021; Qicheng Yang; CAU.

**Figure 21.**
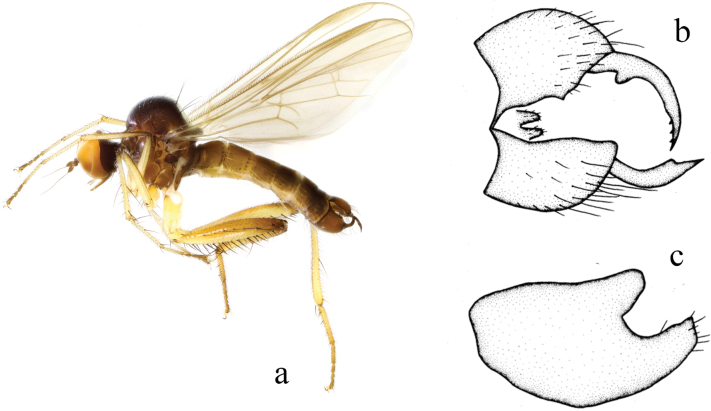
*Hybosserratus***a** male habitus, lateral view **b** genitalia, dorsal view **c** hypandrium, ventral view (from Yang and Yang 1992).

##### Diagnosis.

Legs mostly yellow. Hind tibia with one long ad at middle. Right surstylus curved inward, long hook-like, apically small and acute with three denticles. Hypandrium apically widely incised.

##### Distribution.

China (Zhejiang, Henan, Sichuan, Yunnan, Guizhou, Fujian, Guangxi); Thailand.

#### ﻿ Hybos uniseta

Taxon classificationAnimaliaDipteraHybotidae

Yang & Yang, 2004

C13633F6-BCAB-5039-A5F7-FD5A710B5E85

Fig. 22

Hybos uniseta Yang & Yang, 2004: 221, figs 400–403.

##### Type locality.

China: Zhejiang, Longwangshan.

##### Material examined.

China • 1♂, Fujian, Wuyishan, Jiuquxi; 204 m, 15 April 2021; Ding Yang; CAU.

##### Diagnosis.

Arista short pubescent. Legs mostly yellow, but only hind legs mostly black. Left surstylus bifurcated, right surstylus trifurcated.

**Figure 22.**
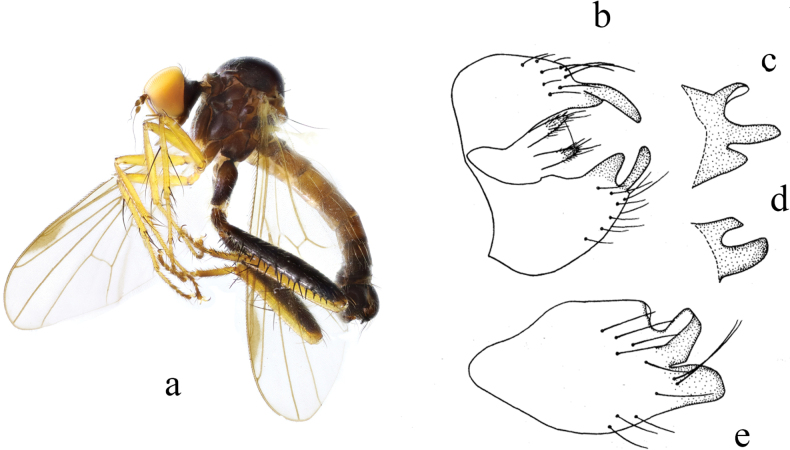
*Hybosuniseta***a** male habitus, lateral view **b** genitalia, dorsal view **c** right surstylus **d** left surstylus **e** hypandrium, ventral view (from Yang and Yang 2004).

##### Distribution.

China (Sichuan, Zhejiang, Fujian).

#### ﻿ Hybos wangae

Taxon classificationAnimaliaDipteraHybotidae

Yang, Merz & Grootaert, 2006

FC5806A0-DAF2-522B-9BC2-9042DE5EE038

Fig. 23

Hybos wangae Yang, Merz & Grootaert, 2006: 803, figs 11–15.

##### Type locality.

China: Guangdong, Nanling.

##### Material examined.

China • 4♂♂, Fujian, Wuyishan, Dazhulan; 935–1,035 m, 3 July 2009; Xiaoyan Liu; CAU. China • 1♂, Fujian, Wuyishan, Liaowangtai, 1,160 m, 14–27 July 2021; Junli Yao (Malaise trap); CAU. China • 1♂, Fujian, Wuyishan, Erlichang, 764 m, 1–8 June 2021; Lingfei Peng (Malaise trap); CAU. China • 1♂, Fujian, Wuyishan, Shibanqiao; 964 m, 8–15 June 2021; Lingfei Peng (Malaise trap); CAU. China • 1♂ 1♀, Fujian, Wuyishan, Fangbanchang, 954 m, 25 May–1 June 2021; Junli Yao (Malaise trap); CAU.

**Figure 23.**
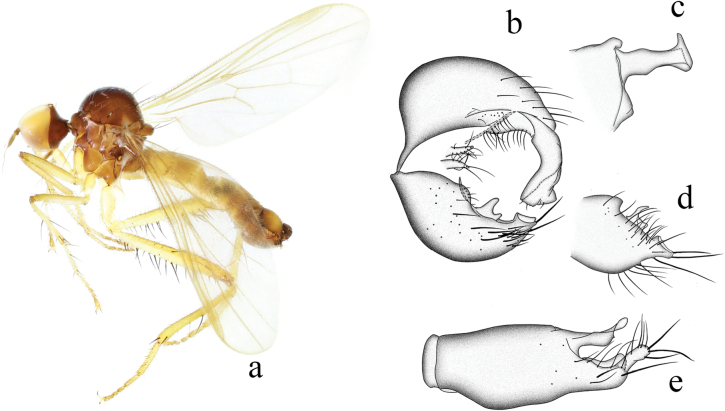
*Hyboswangae***a** male habitus, lateral view **b** genitalia, dorsal view **c** right surstylus **d** left surstylus **e** hypandrium, ventral view.

##### Diagnosis.

Femora yellow except with brown or dark brown apex. Mid tibia with two bristles in dorsal to antero-dorsal position; hind tibia with one ad and one pd at middle. Hypandrium distinctly longer than wide, basally slightly wide, with apico-lateral incision bearing one ridge-like process.

##### Distribution.

China (Fujian, Guangdong).

#### ﻿ Hybos wui

Taxon classificationAnimaliaDipteraHybotidae

Yang & Yang, 1995

8BB98154-ECF9-5386-BA2A-3E366996C14C

Fig. 24

Hybos wui Yang & Yang, 1995: 501, figs 13–15; Yang and Yang 2004: 224, figs 406–408.

##### Type locality.

China: Zhejiang, Baishanzu.

##### Material examined.

China • 1♂ 1♀, Fujian, Wuyishan, Guadun; 1,000–1,200 m, 16 April 2021; Ding Yang; CAU. China • 1♂, Fujian, Wuyishan, Erlichang; 764 m, 10–17 May 2021; Lingfei Peng (Malaise trap); CAU.

**Figure 24.**
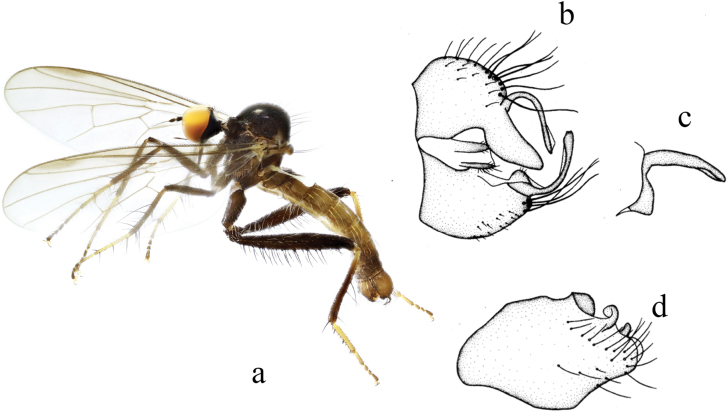
*Hyboswui***a** male habitus, lateral view **b** genitalia, dorsal view **c** left surstylus **d** hypandrium, ventral view (from Yang and Yang 1995).

##### Diagnosis.

Arista short pubescent. Legs mostly black except tarsomeres 1 and 2 dark yellow to yellow. Wing hyaline, stigma indistinct brownish; veins brownish. Left surstylus with one thin finger-like process. Hypandrium short and wide, left lateral margin with one small process apically.

##### Distribution.

China (Zhejiang, Fujian).

#### ﻿ Hybos wuyishanus sp. nov.

Taxon classificationAnimaliaDipteraHybotidae

FBFBF0B6-398E-5340-92BD-2550B094BFAB

https://zoobank.org/4C205536-9EDA-4CE1-986F-C1177B1B7DD4

Fig. 25

##### Type material examined.

***Holotype***: China •♂; Fujian, Wuyishan, Yangjiashan; 1,044 m, 10–17 May 2021; Lingfei Peng (Malaise trap); CAU.

##### Diagnosis.

First flagellomere much elongated, arista long and bare. Legs mostly blackish. Hind tibia with one row of short thin ad and pd. Hypandrium wide at middle, apical margin nearly truncate, with one small process at apical 1/3.

**Figure 25.**
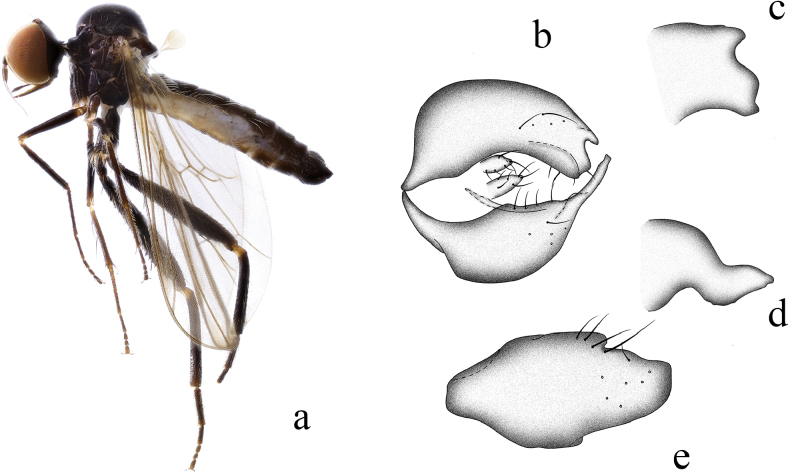
*Hyboswuyishanus* sp. nov. **a** male habitus, lateral view **b** genitalia, dorsal view **c** right surstylus **d** left surstylus **e** hypandrium, ventral view.

##### Description.

Male. Body length 3.8 mm, wing length 3.7 mm.

***Head*** black with gray pilosity. Eyes contiguous on frons, blackish brown with indistinctly enlarged dorsal facets brownish. Hairs and bristles on head blackish except postero-ventral surface with partly brown hairs; ocellar tubercle indistinct with two oc and two short posterior hairs. Antenna blackish; scape without hairs, pedicel with circlet of blackish brown subapical hairs; first flagellomere blackish brown, much elongated, longer than scape and pedicel combined, without dorsal hairs; arista blackish brown, long and bare except apical ~ 1/5 thin. Proboscis shorter than head, blackish brown. Palpus dark brown, with four or five dark brown ventral hairs.

***Thorax*** black with gray pilosity. Hairs on thorax blackish, bristles black; hairs on mesonotum short, ppn absent, two npl (anterior npl weak), uniserial hair-like dc nearly as long as irregularly biserial acr, one prsc, one psa; scutellum with two long sc. Legs mostly blackish, except mid coxa dark brown; hind coxa, femur, and tibia black; all knee brownish yellow; all extreme tip of femur and extreme base of tibiae brownish yellow. Hairs on legs mostly brown to blackish brown, bristles blackish to black, but those on coxae partly brownish yellow; fore femur with dark brown bristles; hind femur and tibia with partly brownish hairs. Fore femur 1.1× and hind femur 2.5× as wide as mid femur. Fore femur with one row of weak pv shorter than femur thickness. Mid femur with three or four ad on apical 1/3, one row of pv (middle pv long, longer than femur thickness). Hind femur with two rows of v, five long av on tubercles and one row of very short spine-like pv on distinct tubercles, apical pv dense and with some very long outer pv hairs. Fore tibia with one long strong preapical ad. Mid tibia with two long strong ad on apical 1/2, one long pv at middle; apically with five bristles including one long preapical pv. Hind tibia with one row of short thin ad and pd. Mid tarsomere 1 with one preapical av. Hind tarsomere 1 with one row of short dense spine-like ventral bristles. Wing hyaline, stigma brown; veins dark brown, R_4+5_ and M_1_ divergent apically. Squama dark yellow with dark yellow hairs. Halter dark yellow with yellow knob.

***Abdomen*** long narrow, nearly straight, blackish with pale gray pilosity. Hairs and bristles on abdomen brownish yellow to dark brown. Hypopygium nearly as thick as pregenital segments.

***Male genitalia*.** Left epandrial lamella slightly narrower than right epandrial lamella, with convex inner margin near middle (Fig. 25b); left surstylus curved, narrowing toward tip (Fig. 25d). Right epandrial lamella with slightly concave inner margin; right surstylus wide with shallow apical incision (Fig. 25c). Hypandrium ~ 1.9× longer than wide, wide at middle, apical margin nearly truncate, with one small process at apical 1/3 (Fig. 25e).

**Female.** Unknown.

##### Etymology.

This specific name refers to the type locality Wuyishan.

##### Distribution.

China (Fujian).

##### Remarks.

The new species is similar to *H.dazhulanus* sp. nov., but may be separated from the latter by the hind tibia with one row of short thin ad and pd and hypandrium with apical margin nearly truncate. In *H.dazhulanus* sp. nov., the hind tibia has one very long thin pd apically, and the hypandrium has an incision at apical margin.

#### ﻿ Hybos xiaohuangshanensis

Taxon classificationAnimaliaDipteraHybotidae

Yang, Gaimari & Grootaert, 2005

ED72FF57-C8B4-5E1F-A05E-6D5A3A08730D

Fig. 26

Hybos xiaohuangshanensis Yang, Gaimari & Grootaert, 2005: 5, figs 9–12.

##### Type locality.

China: Guangdong, Nanling.

##### Material examined.

China • 1♂, Fujian, Wuyishan, Tongmucun, Guadun; 1,033 m, 22 June 2021; Xiaodong Cai; CAU.

**Figure 26.**
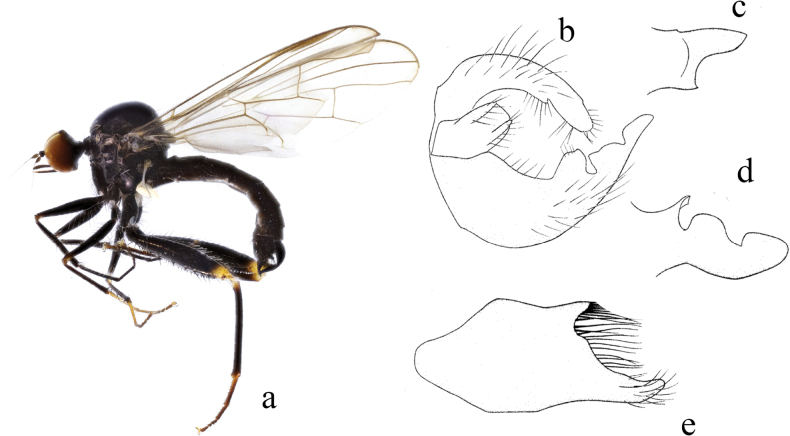
*Hybosxiaohuangshanensis***a** male habitus, lateral view **b** genitalia, dorsal view **c** right surstylus **d** left surstylus **e** hypandrium, ventral view (from Yang et al. 2005).

##### Diagnosis.

Arista bare. Legs black except hind knee and mid and hind tarsi brownish yellow. Hypandrium obliquely incised apically, with long marginal bristles.

##### Distribution.

China (Fujian, Guangdong).

#### ﻿ Hybos zhejiangensis

Taxon classificationAnimaliaDipteraHybotidae

Yang & Yang, 1995

946F9C4B-9708-5350-A6C6-D79F11096DB2

Fig. 27

Hybos zhejiangensis Yang & Yang, 1995: 238, fig. 6; Yang and Yang 2004: 226, figs 413–415.

##### Type locality.

China: Zhejiang, Gutianshan.

##### Material examined.

China • 2♂♂ 3♀♀, Fujian, Wuyishan, Erlichang; 764 m, 10–17 May 2021; Lingfei Peng (Malaise trap); CAU. China • 1♂ 1♀, Fujian, Wuyishan, Liaowangtai; 1,160 m, 10–17 May 2021; Junli Yao (Malaise trap); CAU. China • 1♂ 3♀♀, Fujian, Wuyishan, Xiaonandingkeng; 1,000 m, 25 May–1 June 2021; Junli Yao (Malaise trap); CAU. China • 2♂♂ 3♀♀, Fujian, Wuyishan, Liaowangtai; 1,160 m, 19 April–27 July 2021; Junli Yao (Malaise trap); CAU. China • 3♂♂ 2♀♀, Fujian, Wuyishan, Wulichang; 825 m, 8–15 June 2021; Junli Yao (Malaise trap); CAU. China • 1♂ 2♀♀, Fujian, Wuyishan, Fangbanchang; 954 m, 10–17 May 2021; Junli Yao (Malaise trap); CAU. China • 1♂ 1♀, Fujian, Wuyishan, Erlichang; 764 m, 10–17 May 2021; Lingfei Peng (Malaise trap); CAU.

**Figure 27.**
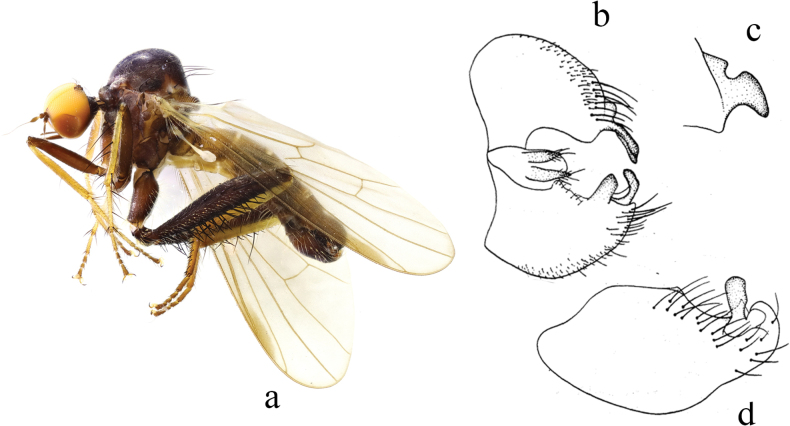
*Hyboszhejiangensis***a** male habitus, lateral view **b** genitalia, dorsal view **c** right surstylus **d** hypandrium, ventral view (from Yang and Yang 1995).

##### Diagnosis.

Arista short pubescent. Legs black except tibiae and tarsi yellow to dark yellow. Hind tibia with two bristles at middle and one lateral bristle near apex. Left surstylus bifurcated.

##### Distribution.

China (Zhejiang, Fujian).

## ﻿Discussion

### ﻿Habitat preferences

Wuyishan National Park belongs to the typical mid-subtropical monsoon climate and is the largest and best-preserved forest ecosystem in the mid-subtropical zone at the same latitude in the world. Huanggang Mountain and Zhumugang Mountain have developed typical altitudinal vegetation belt. The basal belt is the evergreen broad-leaved forest. With the elevation rising, bamboo forest, deciduous broad-leaved forest, temperate coniferous forest, elfin wood, and montane meadow can be seen in turn. The area of evergreen broad-leaved forest accounts for more than 20% of the total forest area, mainly distributed at an altitude of 200 meters to 1,500 meters. The tall mountains in the northwest form a natural barrier, which weakens the invasion of cold air from the north in winter, intercepts the southeast ocean monsoon in summer, and forms a warm and humid monsoon climate in the mid-subtropical region, characterized by significant vertical changes in climate, warm and humid, four distinct seasons, and abundant precipitation (Li 2023). It has dense vegetation and abundant rainfall, with an average annual precipitation of 1,684–1,780 millimeters and an average relative humidity of 78%. The main peak, Huanggang Mount, is 2,158 meters above sea level. We have set up sampling points within the range of 200–1,900 m above sea level, but the *Hybos* specimens obtained are mainly concentrated in 500–1,000 m. All specimens were collected using sweeping and Malaise trap. Most adults were found resting on low vegetation in damp shady places. The research here only focuses on the limited area of Wuyishan National Park, and the new species reported in this work show how little is known about *Hybos*. The collection and investigation in more areas of China are expected to further enrich the species of this genus.

### ﻿Endemicity

The fauna of China has been divided into seven ecoregions: Northeast China, North China, Mongolia-Xinjiang, Qinghai-Tibet, Southwest China, Central China, and South China regions. Wuyishan National Park is located in Fujian Province, which belongs to the eastern part of Central China region. In the zoogeographical division of China, there are six species of *Hybos* in Wuyishan National Park. *Hybosserratus* is a widespread species that is widely distributed in China and has been reported in Thailand. Approximately 48.1% and 25.9% species are from central China and from both Central and South China regions, respectively. In addition, there are species from both Central and North China regions, from both Central and Southwest China regions, and from Central, North, and Southwest China regions. Based on cross-regional comparisons, it can be seen that Central China region has the strongest association with South China region, followed by relatively strong associations with Southwest China and North China regions, and weaker associations with Northeast China, Mongolia-Xinjiang, and Qinghai-Tibet regions.

### ﻿Species identification

Color is often used as one of the main distinguishing features for identifying *Hybos*, but this is only applicable to a few species, and color variation is common in many insect groups. In addition, the presence or absence of bristles on legs is used for identification, but bristles are an unstable features that can be easily removed and their potential impact on species identification should be fully considered. Shapes of the hind leg and veins are sometimes used for species identification; however, with the increasing number of species and specimens, differences between species decrease and individual differences increase, making it very difficult to use these features for species identification. Some scholars use components of the female genitalia to provide information for *Hybos* classification (Plant 2013). Female genitalia of this genus are thought to be more useful for distinguishing between different species than for identifying new ones. If female genitalia are to be used for species identification, a comparative study of female genitalia from all species is necessary. But so far, the females of many species have not been studied and species are only described based on males. Undoubtedly, the components of the female terminalia will provide phylogenetic information for solving the systematic problems of *Hybos*. *Hybos* identification using DNA barcoding and geometric morphology should also be attempted, which is also one of the future research directions.

## Supplementary Material

XML Treatment for Hybos anae

XML Treatment for Hybos ancistroides

XML Treatment for Hybos basiflavus

XML Treatment for Hybos bispinipes

XML Treatment for Hybos brevidigitatus

XML Treatment for Hybos chinensis

XML Treatment for Hybos concavus

XML Treatment for Hybos constractus

XML Treatment for Hybos curvitibia

XML Treatment for Hybos dazhulanus

XML Treatment for Hybos flaviscutellum

XML Treatment for Hybos fujianensis

XML Treatment for Hybos griseus

XML Treatment for Hybos gutianshanus

XML Treatment for Hybos jianyangensis

XML Treatment for Hybos leucopogus

XML Treatment for Hybos longidigitatus

XML Treatment for Hybos longshengensis

XML Treatment for Hybos modificatus

XML Treatment for Hybos orientalis

XML Treatment for Hybos serratus

XML Treatment for Hybos uniseta

XML Treatment for Hybos wangae

XML Treatment for Hybos wui

XML Treatment for Hybos wuyishanus

XML Treatment for Hybos xiaohuangshanensis

XML Treatment for Hybos zhejiangensis

## ﻿Additional information

### Author ORCIDs

Meilin Li https://orcid.org/0000-0003-2473-110X

Ding Yang https://orcid.org/0000-0002-7685-3478

### Data availability

All of the data that support the findings of this study are available in the main text.
